# Stochastic Induction of Long-Term Potentiation and Long-Term Depression

**DOI:** 10.1038/srep30899

**Published:** 2016-08-03

**Authors:** G. Antunes, A. C. Roque, F. M. Simoes-de-Souza

**Affiliations:** 1 Laboratory of Neural Systems (SisNe), Department of Physics, Faculdade de Filosofia Ciências e Letras de Ribeirão Preto, Universidade de São Paulo, Ribeirão Preto, SP, Brazil; 2 Center for Mathematics, Computation and Cognition, Federal University of ABC, São Bernardo do Campo, SP, Brazil

## Abstract

Long-term depression (LTD) and long-term potentiation (LTP) of granule-Purkinje cell synapses are persistent synaptic alterations induced by high and low rises of the intracellular calcium ion concentration ([Ca^2+^]), respectively. The occurrence of LTD involves the activation of a positive feedback loop formed by protein kinase C, phospholipase A_2_, and the extracellular signal-regulated protein kinase pathway, and its expression comprises the reduction of the population of synaptic AMPA receptors. Recently, a stochastic computational model of these signalling processes demonstrated that, in single synapses, LTD is probabilistic and bistable. Here, we expanded this model to simulate LTP, which requires protein phosphatases and the increase in the population of synaptic AMPA receptors. Our results indicated that, in single synapses, while LTD is bistable, LTP is gradual. Ca^2+^ induced both processes stochastically. The magnitudes of the Ca^2+^ signals and the states of the signalling network regulated the likelihood of LTP and LTD and defined dynamic macroscopic Ca^2+^ thresholds for the synaptic modifications in populations of synapses according to an inverse Bienenstock, Cooper and Munro (BCM) rule or a sigmoidal function. In conclusion, our model presents a unifying mechanism that explains the macroscopic properties of LTP and LTD from their dynamics in single synapses.

Long-term depression (LTD) and long-term potentiation (LTP) are persistent activity-dependent modifications of the synaptic strength^1^. One of the best characterized forms of LTD occurs in the synapses between granule cells and Purkinje neurons in the cerebellum^1^. The induction of LTD involves simultaneous stimulations of parallel fibres and climbing fibres at low frequency (~1 Hz), which promote large elevations of the intracellular calcium ion concentration ([Ca^2+^])^1^,^2^, and activate a positive feedback loop formed by protein kinase C (PKC), cytosolic phospholipase A_2_ (PLA_2_), and the extracellular signal-regulated protein kinase (ERK) pathway^3^,^4^. LTD expression results from the phosphorylation of synaptic AMPA receptors (AMPAR_syn_) by PKC^5^,^6^, which disrupts their interactions with the glutamate-receptor interacting protein (GRIP)^7^, and causes their endocytosis^8^.

Granule-Purkinje cells synapses also exhibit postsynaptic LTP induced by repetitive activations of the parallel fibres at low frequency (1 Hz), which cause low Ca^2+^ transients^1^,^2^. LTP involves the activity of protein phosphatase 1 (PP1), protein phosphatase 2A (PP2A), calcineurin (CaN)^9^,^10^, and an increase in the number of AMPAR_syn_^11^.

Experimental findings indicated the existence of specific Ca^2+^ thresholds for the induction of cerebellar synaptic plasticity consistent with the inverse Bienenstock, Cooper and Munro (BCM) rule, which proposes that the postsynaptic strength is potentiated below a sliding modification threshold and depotentiated above it^1^,^12^. However, experiments of photolysis of Ca^2+^-caged compounds demonstrated a sigmoidal correlation between the magnitudes of LTD and the amplitudes of the Ca^2+^ elevations, but failed to obtain LTP^13^. A stochastic computational model of this Ca^2+^-induced LTD demonstrated that its occurrence is probabilistic and modulated by the intensity of the Ca^2+^ transients used as input signals^14^. However, a limitation of this earlier model is that it did not simulate mechanisms implicated with LTP, which could reveal a more complex scenario involving Ca^2+^ and the long-lasting forms of synaptic plasticity.

In this work, we have expanded extensively the previous stochastic computational model of Ca^2+^-induced LTD to simulate LTP and other molecules implicated with LTD. The additional components of the model included Calmodulin (CaM), Ca^2+^/Calmodulin protein kinase II α (αCaMKII)^15^, CaN, Raf kinase inhibitor protein (RKIP), and an endocytic protein (EP) that mediated the internalization of AMPA receptors (AMPARs). Supplementary Table S1 included all the reactions and parameters used in the model and specified which one of them were taken from the previous version. Large-scale computational models of signaling networks are powerful tools for studying the dynamics of the biological systems, but most large-scale models of synaptic plasticity simulate only one process^3^,^16^,^17^. However, the signalling pathways involved with LTP and LTD coexist in the same synapses^1^,^18^ and compete for Ca^2+^ 19. Thus, the aim of our work was to gain insights on the dynamics of the signalling networks involved with the two opposite long-term forms of synaptic plasticity in cerebellum.

Our results showed that, in single synapses, LTD is an all-or-none process, but LTP is graded. Ca^2+^ transients promoted both processes in a stochastic manner. Nevertheless, the intensity of Ca^2+^ signals used to induce synaptic plasticity modulated the likelihood of LTP and LTD occurrences in single synapses. In addition, alterations in the components of the signaling network regulated the effects of Ca^2+^ signals on the induction of LTP and LTD. In consequence, our results indicated the existence of dynamic Ca^2+^ thresholds for the occurrence of macroscopic synaptic modifications according to the inverse BMC rule proposed for cerebellar plasticity^2^. Moreover, by limiting the range of magnitudes of Ca^2+^ transients used as input signals, we obtained the macroscopic sigmoidal relationship observed between the amplitudes of Ca^2+^ signals and the corresponding levels of depression^13^ from the inverse BCM rule. With our novel model, we presented a unifying mechanism of opposite forms of postsynaptic long-term synaptic plasticity in Purkinje cells that described their macroscopic characteristics emerging from their elaborated single synapse dynamics.

## Results

### Stochastic computational model of cerebellar LTD and LTP

**Figure 1:**
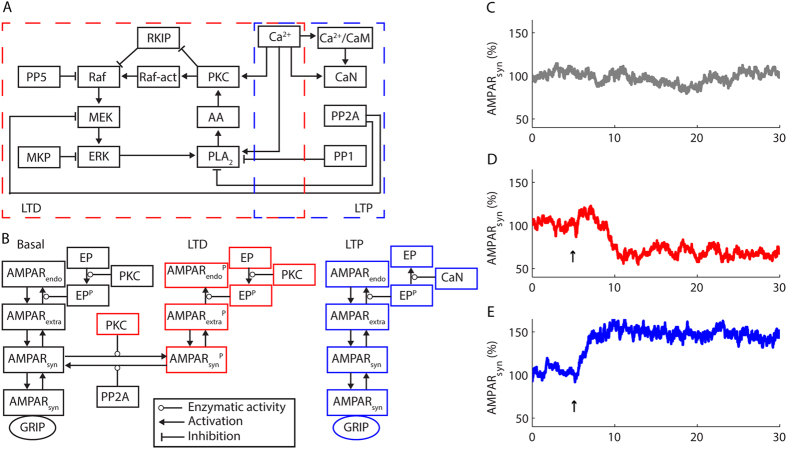
Stochastic computational model of cerebellar LTP and LTD. (**A,B**) Block diagram of the model showing the molecules involved with LTD and LTP (**A**), and the mechanisms of AMPARs trafficking (**B**). At rest, we simulated a constant AMPARs trafficking consisted of lateral diffusion from the synapses (AMPAR_syn_) to extra-synaptic membranes (AMPAR_extra_), from extra-synaptic membranes to endosomes (AMPAR_endo_), and vice-versa. Phosphorylated EP (EP^P^) catalyzed the internalization of AMPAR_extra_. Part of AMPAR_syn_ interacted with GRIP and did not participate in the constant AMPARs trafficking. During LTD, PKC phosphorylated AMPAR_syn_ (AMPAR_syn_^P^) and disrupted their interaction with GRIP promoting their internalization. PP2A counteracted PKC action. During LTP, CaN dephosphorylated EP^P^ and blocked AMPARs internalization. PKC counteracted the action of CaN on EP. (**C**) Simulation of the percentage of AMPAR_syn_ at rest. (**D**) LTD expression consisted of a persistent reduction of AMPAR_syn_. (**E**) During LTP, the model simulated an increase in the percentage of AMPAR_syn_. The arrows indicate LTD and LTP induction with a Ca^2+^ pulse of 3 μmol.L^−1^ and 0.35 μmol.L^−1^, respectively, and 30 s of duration.

The computational model presented in this work simulated LTD and LTP in a single Purkinje cell dendritic spine, which encloses the signalling machinery of the glutamatergic synapse^18^,^20^. We simulated LTD with the positive feedback loop formed by PKC, PLA_2_, and ERK pathway, which we expanded and updated from a previous version^14^ based on early models of synaptic plasticity^3^,^16^. In our model, Ca^2+^ elevations transiently activate PKC and PLA_2_^14^. PKC activates Rapidly accelerated fibrossarcoma (Raf) that activates mitogen-activated ERK kinase (MEK)^21^. MEK activates ERK^21^, which phosphorylates PLA_2_ and sustains its activity after the return of [Ca^2+^] to its basal level^22^. PLA_2_ produces arachidonic acid (AA), a PKC co-factor that activates it synergistically with Ca^2+^ or alone in high concentrations^23^. PKC phosphorylates RKIP and contributes to the activation of Raf^24^. Additionally, PKC regulated ERK pathway through a Raf activator (Raf-act) as implemented previously (Fig. 1A)^14^. The activation of PKC during LTD caused the synaptic depression through the endocytosis of AMPARs^14^. In our simulations, AMPARs were constantly trafficked (diffused, endocytosed and reinserted)^25^ (Fig. 1B,C). However, some synaptic receptors were immobile due to interactions with the scaffold GRIP^7^. The phosphorylation of AMPAR_syn_ by PKC disrupted these interactions^5^,^7^ and promoted their internalization^8^ (Fig. 1B) and the expression of LTD^26^,^27^ (Fig. 1D), which we defined in the model as sustained reductions of the percentage of AMPAR_syn_. We set the basal percentage of AMPAR_syn_ as 100%.

In Purkinje cells, LTP requires protein phosphatases^9^,^10^. PP1 and PP2A^9^, in addition to protein phosphatase 5 (PP5) and the mitogen-activated protein phosphatase (MKP), were included in the signalling network that simulated LTD to counteract the activity of the kinases^14^. During LTP, these phosphatases prevented the activation of the positive feedback loop PKC-PLA_2_-ERK pathway. However, to simulate LTP we had to include CaN in the model (Fig. 1A)^9^,^10^, which we implemented as a heterodimer composed by a CNA subunit that interacts with Ca^2+^/CaM, and a CNB subunit with four Ca^2+^-binding sites^28^,^29^.

CaN is implicated in the trafficking of AMPARs^30^ and plays a pivotal role in endocytosis^31^. The balance between PKC and CaN controls the phosphorylation of dynamin and syndapin, two proteins involved in vesicles endocytosis^31^,^32^. In Purkinje cells, phosphorylated syndapin participates in the endocytosis of AMPARs^8^. The dephosphorylation of syndapin blocks the internalization of AMPARs^8^. Thus, we simulated a protein, which we termed EP, that mediated the internalization of AMPARs, but only in its phosphorylated state^8^. PKC catalysed the phosphorylation of EP^8^. At rest, the model simulated the constant cycle of AMPARs in and out of synapses^25^, which requires the basal activity of PKC^33^ to maintain EP phosphorylated. During LTP, CaN dephosphorylated EP and blocked the endocytosis of AMPARs, without affecting their exocytosis (Fig. 1B). This mechanism of continuous insertion without the concomitant internalization of receptors caused the increase of the AMPAR_syn_ population and the expression of LTP (Fig. 1E). Therefore, the occurrence of LTP in the model involved the persistent increase of the percentage of AMPAR_syn_.

### The role of αCaMKII and phosphatases during the macroscopic occurrence of LTD and LTP

**Figure 2:**
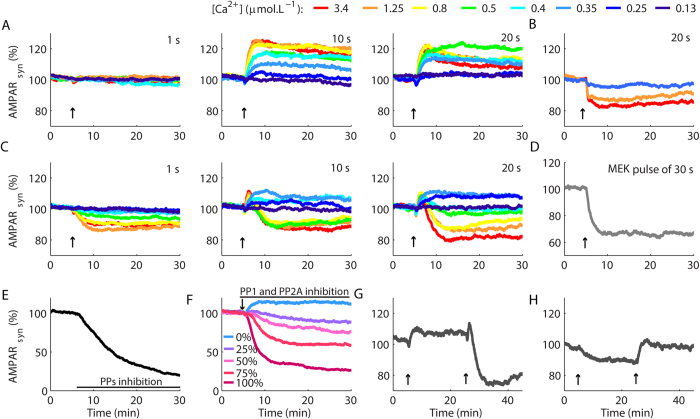
Macroscopic curves of LTP and LTD. (**A**) In the absence of αCaMKII, high amplitude Ca^2+^ pulses of 10 s and 20 s resulted in potentiation. (**B**) The blockage of CaN restored the occurrence of LTD. (**C**) LTP and LTD in the model with αCaMKII. (**D**) A pulse of MEK induced LTD. (**E**) Inhibition of PP1, PP2A and PP5 promoted a slow LTD. (**F**) Gradual blockages of PP1 and PP2A resulted in LTD induction with a protocol of LTP (Ca^2+^ pulse of ~0.4 μmol.L^−1^ and 20 s). (**G,H**) Reversible synaptic plasticity. In (**G**), a Ca^2+^ pulse of ~0.35 μmol.L^−1^ and 20 s induced LTP. After 20 min, a second pulse of 4 μmol.L^−1^ and 20 s induced LTD. In (**H**) we induced weak LTD with a Ca^2+^ pulse of ~0.9 μmol.L^−1^ and 1 s, and restored the basal level of AMPAR_syn_ with a Ca^2+^ pulse of ~0.35 μmol.L^−1^ and 30 s. Each curve in (**A–D**) is the average result of 100 runs of the model, and in (**E–H**) the average of 50 runs. The arrows indicated the occurrence of the Ca^2+^ pulses. We omitted the standard errors of the mean (SEM) for better visualization. The curves with the mean ± SEM are showed in Supplementary Fig. S2.

After the implementation of the model, we used Ca^2+^ pulses with different amplitudes and durations to simulate the photolysis of Ca^2+^-caged compounds, which can induce LTD^13^ and LTP^2^. To compare the results of the simulations with experimental macroscopic curves of LTP and LTD reported in the literature, we used average results of several simulations to represent the responses of populations of synapses. We expected to induce LTP and LTD with Ca^2+^ pulses of low (~0.3 μmol.L^−1^) and high amplitudes (>0.5 μmol.L^−1^), respectively^2^,^13^. Pulses of 1 s of duration caused no change in the synaptic strength (Fig. 2A, Supplementary Figs S1 and S2). Longer pulses (10 s and 20 s) promoted LTP for most amplitudes of Ca^2+^ pulses tested, including high amplitude signals that typically induce LTD in Purkinje cells^1^,^2^,^13^ (Fig. 2A, Supplementary Fig. S1). The blockage of CaN restored the LTD occurrence (Fig. 2B), which indicated that LTP occluded the macroscopic LTD.

Cerebellar LTD requires the activation of the feedback loop PKC-PLA_2_-ERK^3^,^4^, but other molecules are also essential for its occurrence. In αCaMKII knockout mice, protocols of LTD induce LTP in the synapses between granule cells and Purkinje neurons indicating that αCaMKII plays a key role for cerebellar LTD^15^. Thus, to restore LTD without the blockage of CaN, an evident expansion of the model was the inclusion of αCaMKII, a molecule omitted from most models of cerebellar LTD^3^,^13^,^14^.

αCaMKII has several putative targets during synaptic plasticity, including Raf^34^,^35^. Raf activation is a bottleneck for PKC and ERK coupling. Therefore, we implemented αCaMKII acting as a Raf kinase^34^,^35^ (Supplementary Fig. S3). The simulation of αCaMKII comprised its detailed binding to Ca^2+^/CaM and its subsequent autophosphorylation. The autophosphorylation of αCaMKII modulated its affinity for Ca^2+^/CaM and produced an autonomous state that sustained its partial activity^36^ in absence of Ca^2+^/CaM for seconds^37^, but not for hours as classically thought.

αCaMKII inclusion restored the occurrence of macroscopic LTD induced with high Ca^2+^ elevations without affecting LTP induction for low Ca^2+^ transients with prolonged durations (10 s and 20 s) (Fig. 2C). The range of Ca^2+^ rises necessary to induce LTP in the model (0.15–0.4 μmol.L^−1^) was similar to experimental estimations (0.1–0.3 μmol.L^−1^)^2^. We did not observe LTP for stimulations with Ca^2+^ pulses of 1 s, which was consequent to the mechanisms of activation of CaN simulated^38^.

CaN is a heterodimer activated by Ca^2+^ and Ca^2+^/CaM^28^. Under basal [Ca^2+^], two high affinity Ca^2+^-binding sites of the regulatory subunit CNB are constantly filled^29^, but CNA, the subunit that contains the catalytic site of CaN, is inactive^28^,^39^,^40^. The occupancy of the two low affinity Ca^2+^-binding sites of CNB^29^,^41^ during elevations of Ca^2+^ promotes a conformational change that enables the binding of Ca^2+^/CaM to CNA and the exposure of its catalytic site^39^,^40^,^41^. Isolated CNA has low catalytic activity, which is stimulated by Ca^2+^/CaM in absence of CNB^42^,^43^. Nevertheless, in the cells CaN always occurs as the heterodimer CNB/CNA^41^, consequently, its catalytic activation includes the binding of Ca^2+^ to CNB prior to the binding of Ca^2+^/CaM to CNA^28^,^29^,^39^. The binding of Ca^2+^ to CNB occurred with slow rate constants^29^ in our model and limited the activation of CaN for brief signals^38^ impairing LTP induction for Ca^2+^ pulses of 1 s. Consequently, the kinetic aspects of CaN activation constrained the durations of the Ca^2+^ transients^38^ that promoted macroscopic LTP (Fig. 2C).

The direction of the synaptic modifications relies on the balance between the activities of protein kinases and phosphatases^44^. Historically, models of cerebellar LTD implicated the strong inhibition of PP2A by the phosphorylated G-substrate as a key step for synaptic depression^3^,^13^. G-substrate is abundant in Purkinje cells and is a putative target for the nitric oxide (NO)-cyclic guanine monophosphate-dependent protein kinase pathway^45^. However, G-substrate knockout adult mice have normal LTD^45^. Accordingly, we opted to model LTD without the strong inhibition of PP2A simulated previously^3^,^13^. In our model, LTD occurrence involved a shift from a state of low kinase activities at basal [Ca^2+^], to a state of high kinase activities consequent to the activation of the positive feedback loop. Thus, processes that favour the activation of the loop caused LTD in the model. For instance, a pulse of active MEK promoted LTD^4^ (Fig. 2D, Supplementary Fig. S4). In addition, the inhibition of PP1, PP5 and PP2A induced a slow LTD^46^ (Fig. 2E) because it released the inhibition for the activation of the feedback loop PKC-PLA_2_-ERK. Moreover, partial blockages of PP1 and PP2A promoted the induction of LTD for a LTP protocol (Fig. 2F) as observed experimentally^9^, and the magnitudes of the depression varied as a function of the levels of phosphatases inhibition (Fig. 2F), which indicated a concentration-dependent effect. Therefore, the existence of mechanisms of LTP and LTD in the same synapses allowed the model to exhibit different outcomes to equivalent protocols as consequences of alterations in the dynamics of its signalling network.

Another implication of the coexistence of signalling mechanisms of LTP and LTD in the same synapses is the possibility of reversibility of plasticity^1^,^2^. To verify whether our model could exhibit this property, after the induction of LTP with a low and prolonged Ca^2+^ transient, we simulated a strong Ca^2+^ signal and promoted LTD (Fig. 2G). The model also simulated the restoration of the basal synaptic strength after the occurrence of weak LTD (Fig. 2H). However, the model failed to simulate LTP after the induction of strong LTD. The reason for this limitation was the absence of mechanisms to deactivate the positive feedback loop in the model because such mechanisms have not been described. Positive feedback loops promote sustained responses^14^. Consequently, mechanisms that turn off the positive feedback loop PKC-PLA_2_-ERK are crucial for the successive occurrences of opposite forms of synaptic plasticity observed experimentally^2^.

### Stochastic induction of graded LTP and bistable LTD in single synapses

**Figure 3:**
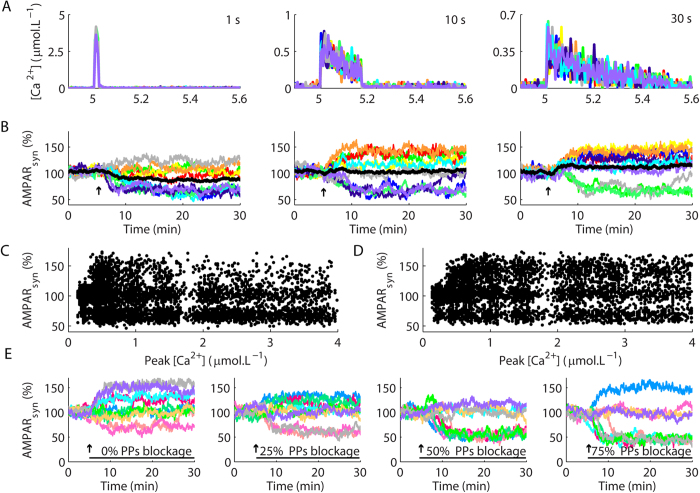
Stochastic induction of LTP and LTD in single synapses. (**A,B**) Ca^2+^ pulses of different peak amplitudes and durations (**A**) induced bistable LTD and graded LTP in the model (**B**). The black lines are the average results calculated for the 10 runs of the model showed with multiple colours in each panel. The result of each single run for each Ca^2+^ pulse duration tested is showed with consistent colours in panels A,B. (**C**) Changes of the percentage of AMPAR_syn_ (measured 25 min after the induction of plasticity) as functions of the peak amplitudes of the Ca^2+^ pulses (Peak [Ca^2+^]) used to promote plasticity. (**D**) Changes of the percentage of AMPAR_syn_ as functions of Peak [Ca^2+^] used to induce synaptic modification in the model without αCaMKII. Each dot in (**C,D**) is the result of a single simulation. The durations of the Ca^2+^ pulses ranged from 1 s to 30 s. (**E**) Effects of the partial blockages of PP1 and PP2A (termed PPs in the panels) in simulations of single synapses stimulated with a protocol used to induce macroscopic LTP (a single Ca^2+^ pulse of ~0.35 μmol.L^−1^ and 20 s of duration).

The signalling machinery involved with plasticity in glutamatergic synapses is located in dendritic spines, small structures that act as isolated biochemical compartments^20^. Each spine encloses a signalling population consisting of few copies of several different molecules^18^ susceptible to undergo high amplitude stochastic fluctuations in their activities^14^. Usually, experimental curves of LTP and LTD represent the macroscopic integration of hundreds to thousands of synapses. In the model, we reproduced the macroscopic curves using average results of several simulations of plasticity in single synapses. However, in signalling systems susceptible to stochasticity, the average behaviour can diverge from unitary events^14^. Thus, the next stage of our work investigated the characteristics of LTP and LTD in single synapses induced by Ca^2+^ pulses with different amplitudes and durations (Fig. 3A).

In single synapses, LTD was an all-or-none process (Fig. 3B), as demonstrated previously^14^. This bistability resulted from the activation of the positive feedback loop PKC-PLA_2_-ERK, which promotes robust and persistent responses^14^. In contrast, LTP happened without mechanisms of self-regulation or amplification. The levels of potentiation resulted from the competition between the activity of CaN and PKC on EP, their common substrate. Consequently, LTP in single synapses was graded (Fig. 3B).

The magnitudes of the Ca^2+^ pulses did not ensure the occurrence of a specific type of plasticity in single synapses. Ca^2+^ signals with equivalent peak amplitudes and durations promoted either the occurrence of LTP and LTD, or failed to induce synaptic modifications (Fig. 3A,B, Supplementary Fig. S5). Therefore, the dynamics of plasticity in single synapses diverged from average responses (Fig. 3B, black lines).

The occurrence of opposite forms of plasticity induced by equivalent Ca^2+^ transients indicated that both LTP and LTD were stochastic processes in the model, which we corroborated assessing the changes of AMPAR_syn_ as a function of the peak Ca^2+^ rises (Fig. 3C). We measured the alterations of AMPAR_syn_ 25 minutes after the induction of plasticity with Ca^2+^ pulses of different durations and peak amplitudes. Our results demonstrated a high rate of LTD, measured as reductions of AMPAR_syn_ from its basal value (set as 100%), for the entire range of Ca^2+^ amplitudes tested. However, LTD predominated as the synaptic modification obtained in the model for stimulations with high amplitude Ca^2+^ transients (>0.8 μmol.L^−1^) (Fig. 3C). In contrast, LTP, verified as increases of AMPAR_syn_ from its basal value, happened preferentially for low Ca^2+^ signals (Fig. 3C).

Next, we addressed whether other components of the model regulated the role of Ca^2+^ on LTP and LTD inductions. Thus, we verified the changes of AMPAR_syn_ as functions of the peak amplitudes of Ca^2+^ transients for simulations performed without αCaMKII. The results showed that the absence of αCaMKII increased the range of Ca^2+^ amplitudes that induced LTP and decreased the occurrence of LTD (Fig. 3D). This increase of LTP occurrence combined with the reduction of LTD occurrence promoted the macroscopic curves of plasticity that failed to exhibit macroscopic depression showed previously (Fig. 2A).

Simulations of plasticity in single synapses induced with a LTP protocol in the presence of partial blockages of PP1 and PP2A indicated that the reduction of phosphatases activities increased the occurrence of microscopic LTD in a concentration-dependent manner (Fig. 3E), and promoted the macroscopic curves with different magnitudes of depression showed in Fig. 2F. Thus, while the absence of αCaMKII increased the induction of LTP (Fig. 3D) in comparison to the control model (Fig. 3C) and disrupted the occurrence of macroscopic LTD (Fig. 2A), reduction of the activities of PP1 and PP2A increased LTD occurrence in single synapses (Fig. 3E) and promoted macroscopic curves of depression for protocols that should induce LTP (Fig. 2F). Historically, the discrimination between the induction of LTP and LTD is attributed to the existence of specific Ca^2+^ thresholds, which would activate Ca^2+^-dependent kinases and phosphatases with distinct Ca^2+^-affinities^19^. Our results demonstrated that different magnitudes of Ca^2+^ signals modulate the stochastic induction of LTP and LTD, but this modulation was not fixed. Changes in the components of the model regulated the role of Ca^2+^ on the induction of LTP and LTD in a dynamic manner.

**Figure 4:**
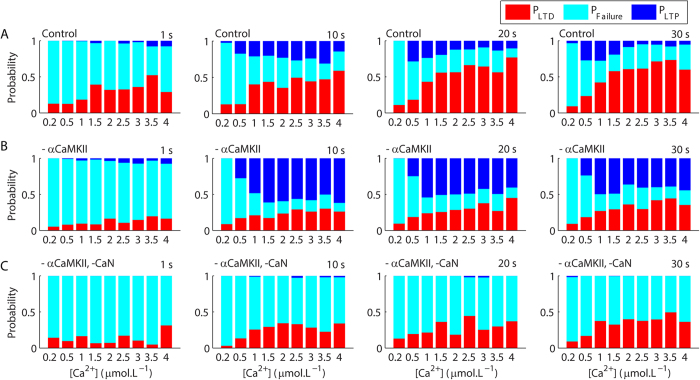
Probabilistic induction of long-term synaptic plasticity. (**A**) The durations and peak amplitudes of the Ca^2+^ pulses regulated the probabilities of induction of LTP (P_LTP_), LTD (P_LTD_), and failure of induction of synaptic plasticity (P_Failure_) in the control model. The regulatory role of Ca^2+^ on P_LTP_ and P_LTD_ was further modulated by the absence of αCaMKII (**B**), which promoted a global reduction of P_LTD_ and an increase of P_LTP_. The simultaneous absence of αCaMKII and CaN had no additional effect on P_LTD_, but suppressed the occurrence of LTP (**C**). For each Ca^2+^ concentration in each panel, we analysed the results of 50–200 single runs of the model.

To quantify the role of Ca^2+^ signals on the induction of LTP and LTD, we calculated the probability of unitary occurrences of LTD (P_LTD_), LTP (P_LTP_) and failure of plasticity (P_Failure_) for Ca^2+^ pulses with different amplitudes and durations. Our results showed that P_LTD_ increased with the increment of the durations and peak amplitudes of the Ca^2+^ signals used to trigger plasticity (Fig. 4A)^14^. P_LTP_ was low for short Ca^2+^ pulses (1 s). For Ca^2+^ signals of 10 s, P_LTP_ was low for weak Ca^2+^ rises, increased for amplitudes ranging from 1–3.5 μmol.L^−1^ and dropped for higher concentrations while P_LTD_ increased progressively with the increment of the amplitudes of the Ca^2+^ signals (Fig. 4A). For pulses of 20–30 s of duration, P_LTP_ was high for Ca^2+^ signals with low peak amplitudes (0.5–1 μmol.L^−1^), and decreased progressively with the increment of the amplitudes of the pulses, which caused the increase of P_LTD_ (Fig. 4A). Taken together, these results suggested that the activation of the Ca^2+^-dependent molecules involved with both LTP and LTD increased with the intensification of the magnitudes of the Ca^2+^ signals. However, because cerebellar LTD involves the activation of a positive feedback loop, which produces sustained patterns of activation^14^, its occurrence occluded LTP. To investigate this hypothesis, we calculated P_LTD_, P_LTP_ and P_Failure_ for modified versions of the model. Simulations performed in absence of αCaMKII presented a clear reduction of P_LTD_ in comparison with the control model and higher P_LTP_ for all magnitudes of Ca^2+^ signals tested (Fig. 4B). In contrast, simulations of the model in the absence of αCaMKII and CaN (Fig. 4C) had no LTP, but exhibited P_LTD_ similar to the values observed for the model without αCaMKII (Fig. 4B). These results indicated that the occurrence of LTP did not interfere with P_LTD_ in the conditions tested, but LTD occurrences altered P_LTP_. Therefore, the components of the model affected in a non-linear manner the occurrences of unitary LTP and LTD and dynamically regulated the role of Ca^2+^ on the stochastic induction of the opposite forms of synaptic plasticity.

### Macroscopic Ca^2+^ thresholds to induce LTD and LTP

**Figure 5:**
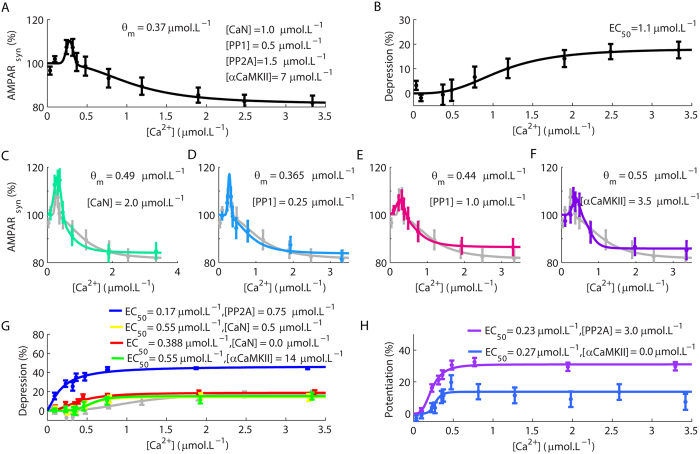
Macroscopic Ca^2+^ thresholds for the occurrences of synaptic modifications. (**A,B**) An inverse BCM rule (**A**) and a sigmoid function (**B**) described the correlation between the macroscopic synaptic modifications and the magnitudes of Ca^2+^ pulses according to the range of Ca^2+^ amplitudes analysed. The term EC_50_ stands for the [Ca^2+^] required to achieve half-maximum LTD. (**C**) Simulations performed with higher [CaN] (the control concentration of CaN and other components of the model are indicated in (**A**)). (**D,E**) A reduction of [PP1] (**D**) had little effect on the overall behaviour of the model, but an increase (**E**) in its concentration changed *θ*_*m*_. (**F**) Reduction of [αCaMKII] altered the value of *θ*_*m*_ of the inverse BMC rule. In (**C–F**) the light gray line corresponds to the results obtained with the control model (**A**) replotted for comparison. (**G**) An increase of [αCaMKII] or a reduction of [CaN] or [PP2A] suppressed the occurrence of LTP (the control result (**B**) was replotted in gray for comparison). (**H**) The elevation of [PP2A] or the absence of αCaMKII resulted in the suppression of LTD. Each dot in the panels is the mean + SEM calculated for 100 simulations. The duration of the Ca^2+^ pulses was 20 s for all simulations.

The modulatory role of Ca^2+^ transients in the directions of synaptic plasticity in single synapses determined macroscopic Ca^2+^ thresholds for the induction of LTD and LTP (Fig. 5A, Supplementary Fig. S6). These results were observed with Ca^2+^ pulses of 10–30 s. In Fig. 5A, the first Ca^2+^ threshold, observed for low amplitude Ca^2+^ transients, represented the change from non-plasticity to LTP, and the second was the threshold for the conversion of LTP to LTD, which corresponds to the crossover point (*θ*_*m*_) predicted in the classical BCM rule^12^. Thus, the macroscopic curves of plasticity obtained with our model corroborated the existence of Ca^2+^ thresholds for synaptic modifications consistent with the inverse BMC rule^2^.

Interestingly, a previous work reported the existence of a macroscopic sigmoidal relationship between the magnitudes of LTD and the levels of Ca^2+^ rises, but failed to observe LTP^13^. However, the range of [Ca^2+^] investigated in this work varied from approximately 0.5 to 6 μmol.L^−1^ 13. In our work, we observed macroscopic LTP only for Ca^2+^ transients lower than 0.5 μmol.L^−1^ (*θ*_*m*_ = 0.37 μmol.L^−1^), which is consistent with other experimental results^2^. Nevertheless, by removing the results obtained for Ca^2+^ pulses below from 0.5 μmol.L^−1^ from our analyses, we obtained the same sigmoid function observed experimentally^13^ (Fig. 5B). Therefore, our results supported the existence of the two macroscopic rules (the inverse BCM rule and the sigmoid relationship) to describe the relations between the levels of Ca^2+^ rise and the occurrence of opposite forms of synaptic plasticity.

In the simulations of single synapses described previously (Figs 3 and 4), we verified that different components of the model affected the occurrences of plasticity. These results suggested that the components of the model regulate the macroscopic Ca^2+^ thresholds for LTP and LTD. To investigate this aspect of the model, we varied the concentrations of some of its components and verified their impacts on the macroscopic Ca^2+^ thresholds for the induction of LTP and LTD (Fig. 5C–H, Supplementary Fig. S7). Simulations with a higher concentration of CaN ([CaN] = 2 μmol.L^−1^, the control concentration was 1 μmol.L^−1^) resulted in stronger LTP and affected *θ*_*m*_ (Fig. 5C). A reduction of PP1 concentration ([PP1] = 0.25 μmol.L^−1^, in the control model [PP1] = 0.5 μmol.L^−1^) had no effect on the Ca^2+^ thresholds for LTP and LTD, but an increase of [PP1] (1 μmol.L^−1^) altered *θ*_*m*_ (Fig. 5D,E). We also observed an alteration of *θ*_*m*_ for simulations with a reduced αCaMKII concentration ([αCaMKII] = 3.5 μmol.L^−1^, [αCaMKII] was ~7 μmol.L^−1^ in the control model) (Fig. 5F). In contrast, an increase of [αCaMKII] (14 μmol.L^−1^) suppressed LTP and promoted a sigmoidal relation between the amplitudes of the Ca^2+^ signals and the magnitudes of LTD (Fig. 5G). We verified similar results for simulations with reduced [CaN] (0.5 and 0 μmol.L^−1^) and reduced concentration of PP2A ([PP2A] = 0.75 μmol.L^−1^, its control concentration was 1.5 μmol.L^−1^) (Fig. 5G). Additionally, all these curves (Fig. 5G) exhibited lower Ca^2+^ requirement to achieve half-maximum depression (EC_50_) in comparison to the control model (Fig. 5G light gray line). We suppressed LTD and obtained sigmoidal relations between the amplitudes of the Ca^2+^ signals and the magnitudes of LTP by setting [αCaMKII] as 0 μmol.L^−1^ or increasing [PP2A] (from 1.5 μmol.L^−1^ to 3 μmol.L^−1^) (Fig. 5H). Therefore, alterations of the molecules involved with synaptic plasticity affected the thresholds and the rules that associate changes of [Ca^2+^] with the directions of the macroscopic forms of synaptic plasticity.

**Figure 6:**
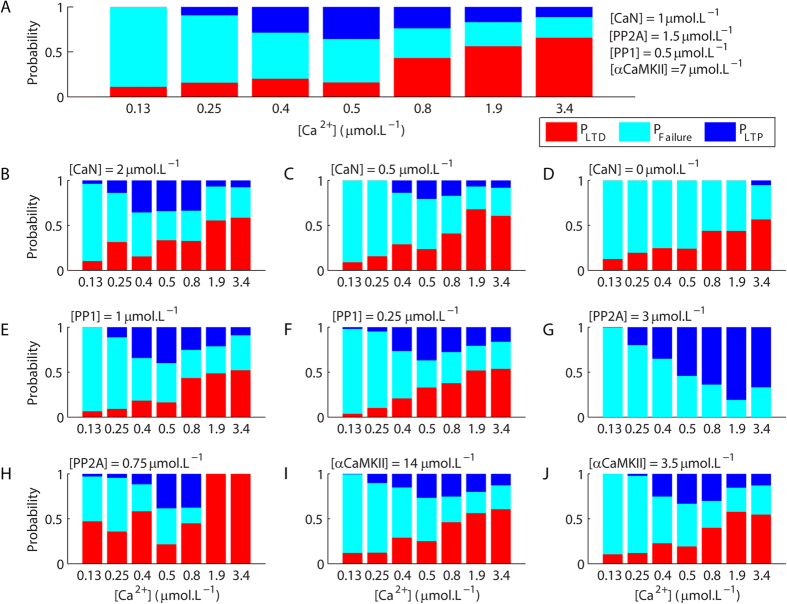
Modulations of the probabilities of LTP and LTD inductions. (**A**) Probabilities of LTP (P_LTP_), LTD (P_LTD_), and failure (P_Failure_) of inductions of synaptic plasticity for the control model stimulated with Ca^2+^ pulses of 20 s of duration and different peak concentrations. (**B**–**J**) P_LTP_, P_LTP_, and P_Failure_ obtained for Ca^2+^ pulses of 20 s for modified versions of the model with an increase of [CaN] (**B**), decrease of [CaN] (**C**), absence of CaN (**D**), increase of [PP1] (**E**), decrease of [PP1] (**F**), increase of [PP2A] (**G**), decrease of [PP2A] (**H**), increase of [αCaMKII] (**I**), and decrease of [αCaMKII] (**J**). For each Ca^2+^ concentration in each panel, we analysed the results of 50–100 single runs of the model.

Microscopically, the curves presented in Fig. 5 emerged from the combinations of the probabilities of LTP and LTD occurrences and the probability of failure of synaptic plasticity. The BCM rule observed for the control model (Fig. 5A) resulted from the balance of P_LTP_, P_LTD_ and P_Failure_ that changed with the amplitudes of the Ca^2+^ signals used to induce plasticity (Fig. 6A). Modifications of the components of the model that promoted alterations in the rules and thresholds for the macroscopic forms of synaptic plasticity did so by affecting the balances between P_LTP_, P_LTD_ and P_Failure_. The increase of [CaN] that promoted stronger LTP and affected *θ*_*m*_ in Fig. 5C resulted from an overall increase of P_LTP_ and from alterations in the peak amplitudes of the Ca^2+^ signals associated with maximum P_LTP_ (Fig. 6B). Decreases of [CaN] had the opposite effect (Fig. 6C,D). The increase of [PP1] caused an enhancement of P_LTP_ for some amplitudes of Ca^2+^ transients (Fig. 6E), and the decrease of [PP1] had little effect on both P_LTP_ and P_LTD_ (Fig. 6F), which is consistent with the fact that this alteration had only slight effects on the Ca^2+^ thresholds for macroscopic LTP and LTD occurrences (Fig. 5D). In contrast, the increase of [PP2A] suppressed the occurrence of unitary LTD (P_LTD_ = 0). As a result, the balance between P_Failure_ and P_LTP_ (Fig. 6G) promoted a sigmoid function between the levels of Ca^2+^ rises and the magnitudes of macroscopic LTP (Fig. 5H). The reduction of [PP2A] had the opposite effect and decreased P_LTP_ and enhanced P_LTD_ (Fig. 6H). The increase of [αCaMKII], which blocked the occurrences of macroscopic LTP (Fig. 5G), did not supress the occurrence of unitary LTP, but decreased P_LTP_ (Fig. 6I). However, the decrease of [αCaMKII] altered P_LTP_ and P_LTD_ for intermediary peak amplitudes of the Ca^2+^ signals (Fig. 6J) in comparison to the control model and, in consequence, affected *θ*_*m*_ of the macroscopic LTP and LTD occurrences (Fig. 5F). Thus, the macroscopic relations between Ca^2+^ and the inductions of LTP and LTD are dynamically regulated by changes in the probabilities of unitary occurrences of synaptic plasticity.

## Discussion

We presented a unifying model of postsynaptic cerebellar LTP and LTD in Purkinje cells. There are few other models of the signalling mechanisms of synaptic plasticity in the cerebellum, and most of them focused only on LTD and were solved deterministically^3^,^13^,^47^. The first stochastic model of LTD is recent and indicated that stochasticity plays a central role in the macroscopic curves of plasticity^14^. In this work, we have expanded and updated the stochastic model of LTD and incorporated mechanisms to simulate LTP. The model reproduced several properties of LTP and LTD observed experimentally, and correlated them with the dynamics of plasticity in single synapses. In this way, the model provided a unified mechanistic explanation for many experimental observations of LTP and LTD occurrences in granule-Purkinje cell synapses. Still, the model has important limitations. For instance, experimental findings reported that both NO and derivatives of AA are involved with LTP and LTD^11^,^48^, and CaMKII regulates NO at least during the depression^47^, but these processes were not implemented in our model. Nevertheless, our work revealed new aspects of the dynamics of LTD and LTP that are testable experimentally.

Historically, the balance between the activity of protein kinases and phosphatases regulated by Ca^2+^ is considered the key element for the discrimination between the occurrences of LTP and LTD^19^,^44^. This observation was initially proposed for the synapses between CA3 and CA1 hippocampal pyramidal neurons^19^. Hippocampal LTP involves the activation of αCaMKII, and LTD requires CaN^19^,^44^. Both CaN and αCaMKII are activated by Ca^2+^/CaM^39^,^41^,^49^, but CaN has a 1000-fold higher affinity for Ca^2+^/CaM in comparison to αCaMKII (~12 pmol.L^−1^ 50 for CaN and ~4–20 nmol.L^−1^ for αCaMKII, which corresponds to its affinity measured in presence of nucleotides^51^,^52^,^53^). Hippocampal LTD and LTP require low and high Ca^2+^ rises, respectively^54^. Consequently, a central hypothesis to explain the direction of the synaptic plasticity explored in many computational models^17^,^55^ relies on the differences between the affinities of CaN and αCaMKII for Ca^2+^/CaM, which would promote their differential activations for the low and high Ca^2+^ elevations required for hippocampal LTD and LTP induction, respectively^19^. However, our results indicated that the extrapolation of this idea to the discrimination between LTP and LTD for different levels of Ca^2+^ rises in Purkinje cells is an oversimplification.

In this work, unitary occurrences of LTP and LTD were stochastic processes. LTD was bistable, but LTP was graded. Due to the probabilistic nature of unitary LTP and LTD, the amplitudes of Ca^2+^ elevations used as input signals did not ensure the occurrence of any particular type of plasticity and played only a modulatory role. Additionally, both LTP and LTD were highly modulated by other signalling species of the model. Thus, not only CaN and αCaMKII, but also several other components of the model affected the probabilities of unitary occurrences of LTP and LTD. Consequently, our results support the existence of dynamic rather than static macroscopic Ca^2+^-thresholds for the occurrences of LTP and LTD.

## Materials and Methods

We built the computational model of LTP and LTD using BioNetGen^56^, a rule-based software for modelling biochemical networks. We solved the simulations stochastically with the SSA algorithm.

The model consisted of a well-mixed compartment containing mechanisms of Ca^2+^ dynamics, the signalling network involved with LTP and LTD, and AMPARs trafficking. The detailed descriptions of the components of the model with their respective parameters (Supplementary Table S1), references, validations (Supplementary Fig. S8), and additional analyses (Supplementary Fig. S9) are given in the Supplementary Methods.

Most simulations modelled a time interval of 37 minutes; the first seven minutes comprised the period necessary for the system to reach steady-state and were withdrawn from the analyses. The time course analysed included an initial interval of five minutes before the inductions of plasticity plus 25 minutes, a temporal interval in which the activation of the feedback loop is essential^4^. In Figs 3C,D and 5, we measured the percentage of AMPAR_syn_ 25 minutes after the induction of plasticity. In Fig. 5, the dots in each curve are means ± standard error of the mean (SEM) calculated for 100 simulations.

We fitted the curves of Fig. 5A,C–F using an equation given as follows:


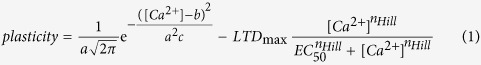


where *a* is a scaling factor, *b* refers to the [Ca^2+^] required for maximum LTP, *c* stands for the width of the Gaussian curve for LTP occurrence, *LTD*_max_ refers to the maximum depression, *n*_*Hill*_ is the Hill coefficient and *EC*_*50*_ is the [Ca^2+^] required to induce half-maximum depression.

The sigmoid function used to fit the curves of Fig. 5B,G is described as (15):


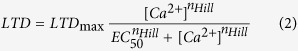


We used the same Equation (2) to fit the sigmoid curves of Fig. 5H replacing LTD for LTP. We fitted all curves in Fig. 5 using the Matlab Curve Fitting Tool (cftool) with 95% of confidence interval.

To calculate the probabilities of LTP and LTD induction in Figs 4 and 6, we measured the percentage of AMPAR_syn_ 25 minutes after the induction of synaptic plasticity for single runs of the model stimulated with Ca^2+^ pulses with different durations and peak amplitudes. We defined increases of AMPAR_syn_ of 20% and above as LTP and reductions of AMPAR_syn_ of 20% and below as LTD. Variations of the population of AMPAR_syn_ between 80–120% (the standard population was set as 100%) were attributed to the stochasticity of the model and treated as failures of synaptic plasticity induction.

## Additional Information

**How to cite this article**: Antunes, G. *et al*. Stochastic Induction of Long-Term Potentiation and Long-Term Depression. *Sci. Rep*. **6**, 30899; doi: 10.1038/srep30899 (2016).

## Supplementary Material

Supplementary Information

Supplementary Dataset 1
