# Supplementary material for: Stochastic Induction of Long-Term Potentiation and Long-Term Depression
Source: Sci Rep. 2016 Aug 3;6:30899. doi: 10.1038/srep30899 (PMC4971485; doi:10.1038/srep30899)
Supplement: Supplementary Information [file srep30899-s1.pdf]

Supplementary material

Title: Stochastic Induction of Long-Term Potentiation and Long-Term Depression

Authors: G. Antunes[1], A. C. Roque[1], F. M. Simoes-de-Souza[2]*

Affiliations:

[1]Laboratory of Neural Systems (SisNe), Department of Physics, Faculdade de Filosofia Ciências e Letras de Ribeirão Preto, Universidade de São Paulo, Ribeirão Preto, SP, Brazil

[2]Center for Mathematics, Computation and Cognition, Federal University of ABC, São Bernardo do Campo, SP, Brazil

*To whom correspondence should be addressed: fabio.souza@ufabc.edu.br

Supplementary Fig. S1. Ca^{2+} pulses used as input signals

Supplementary Fig. S2. Replots of the curves showed in Fig. 2 with standard error bars

Supplementary Fig. S3. Block diagram of the model with αCaMKII

Supplementary Fig. S4. Activation of the signalling network during MEK-induced LTD and Ca^{2+}-induced LTD

Supplementary Fig. S5. Activations of the components of the model during LTP and LTD in single synapses

Supplementary Fig. S6. Curves of synaptic modifications as functions of Ca^{2+} elevations with different durations

Supplementary Fig. S7. Time courses of synaptic plasticity for altered signalling networks

Supplementary Fig. S8. Dose-response curves

Supplementary Fig. S9. Sensitivity analyses

Supplementary Methods

Supplementary Table S1. Parameters of the model

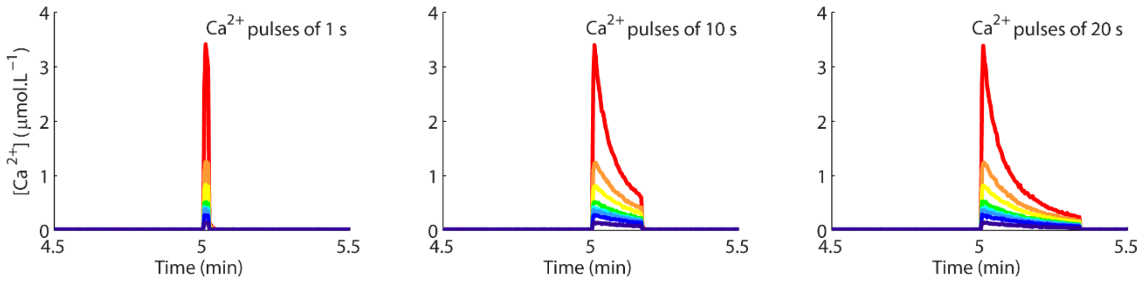

Supplementary Fig. S1. Ca^{2+} pulses used as input signals. These pulses were the input signals for the simulations showed in Fig. 2A. The shape of the pulses resulted from the Ca^{2+} dynamics implemented. Each curve is the average result of 100 single runs of the model.

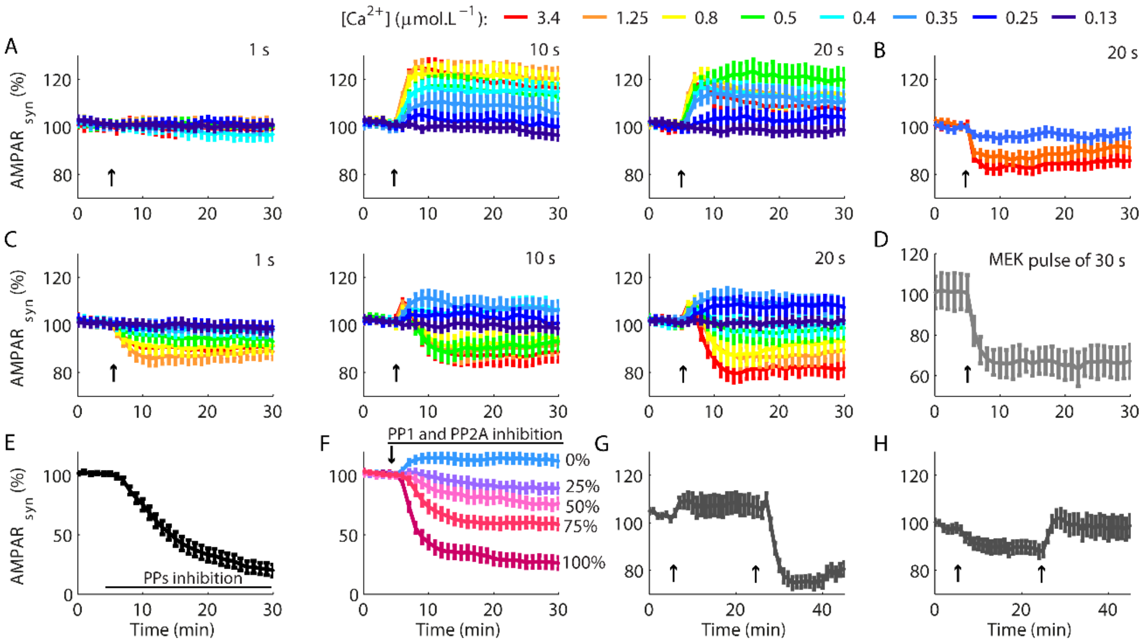

Supplementary Fig. S2. Replots of the curves showed in Fig. 2 with standard error bars. Each curve represents mean ± standard error of the mean calculated from 100 (A-D) or 50 (E-H) single runs of the model.

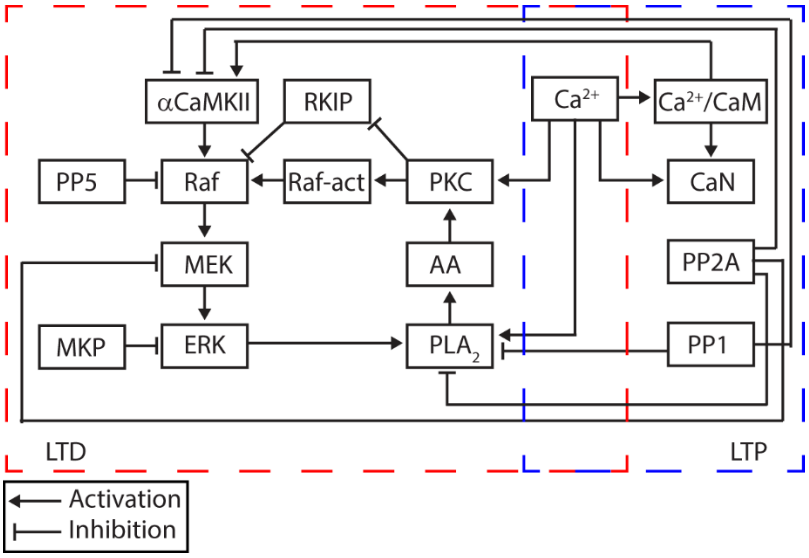

Supplementary Fig. S3. Block diagram of the model with αCaMKII. During LTD, αCaMKII acted as a Raf kinase.

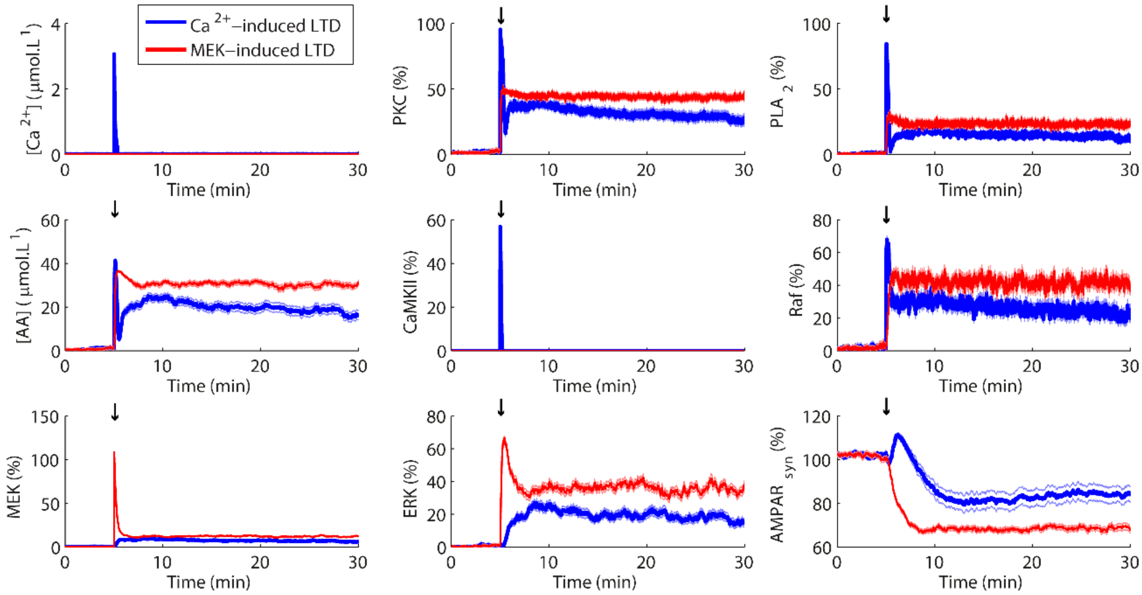

Supplementary Fig. S4. Activation of the signalling network during MEK-induced LTD and Ca^{2+}-induced LTD. A pulse of activated MEK (30 s of duration) induced a strong depression in the model through the activation of the positive feedback loop formed by PKC, PLA_2, and ERK pathway, without the activation of αCaMKII. Each curve represents mean (bold lines) $\pm$ standard error of the mean (light lines) from 50 single runs of the model. The arrows indicate the moment of occurrence of the pulse of stimulation.

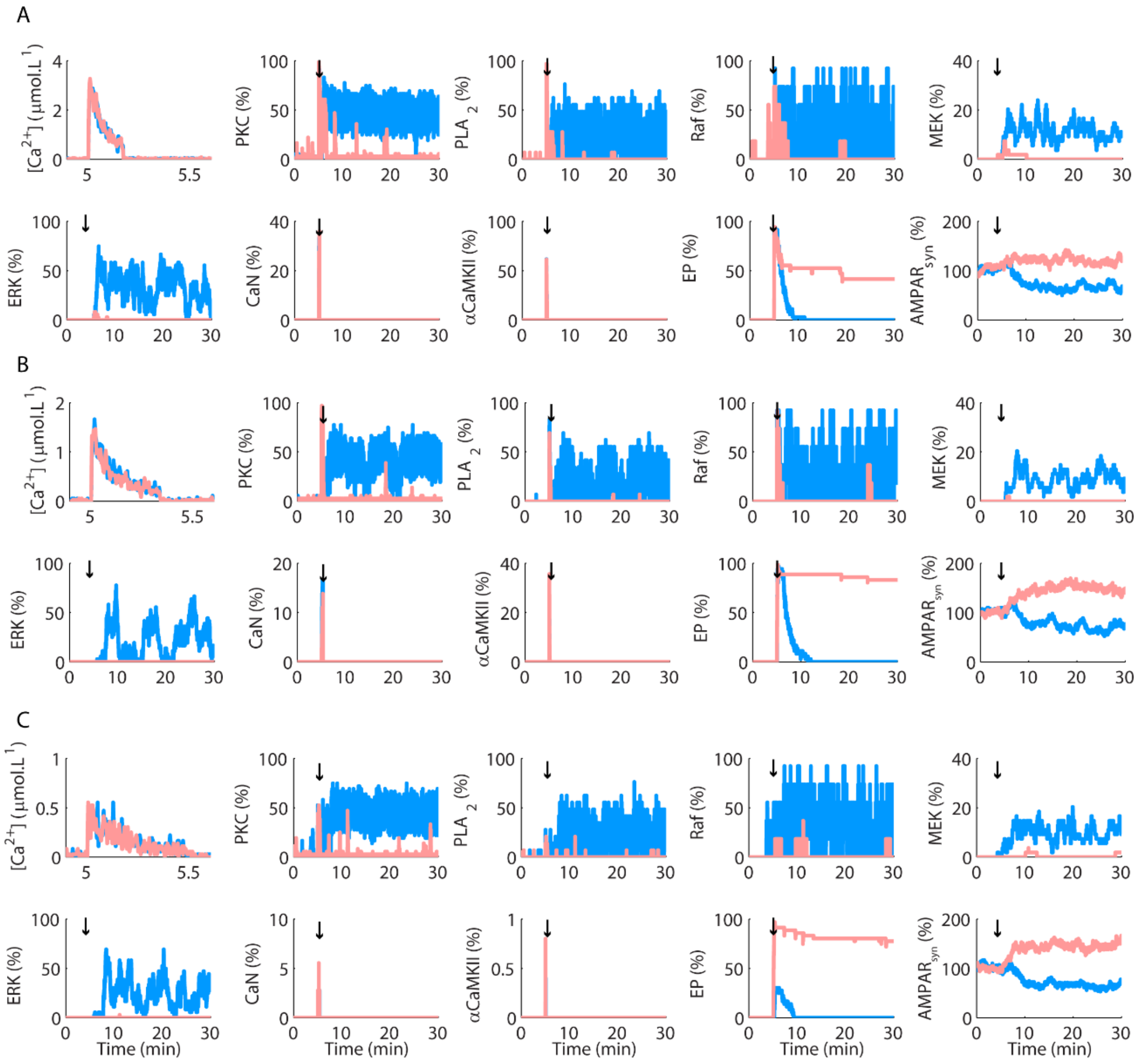

Supplementary Fig. S5. Activations of the components of the model during LTP and LTD in single synapses. (**A-C**) Time courses of the activities of the components of the model during LTD (light blue lines) and LTP (light pink lines) induced with Ca^{2+} pulses of 10 s (**A**), 20 s (**B**), and 30 s **(C)** of duration. The arrows indicate the instant of the occurrence of the Ca^{2+} pulses.

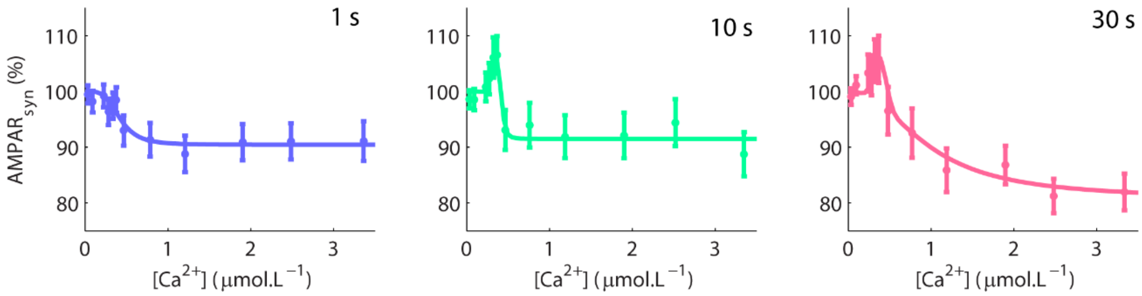

Supplementary Fig. S6. Curves of synaptic modifications as functions of Ca^{2+} elevations with different durations. The durations are indicated in the panels. Each dot is the mean $\pm$ standard error of the mean calculated for 100 runs of the model.

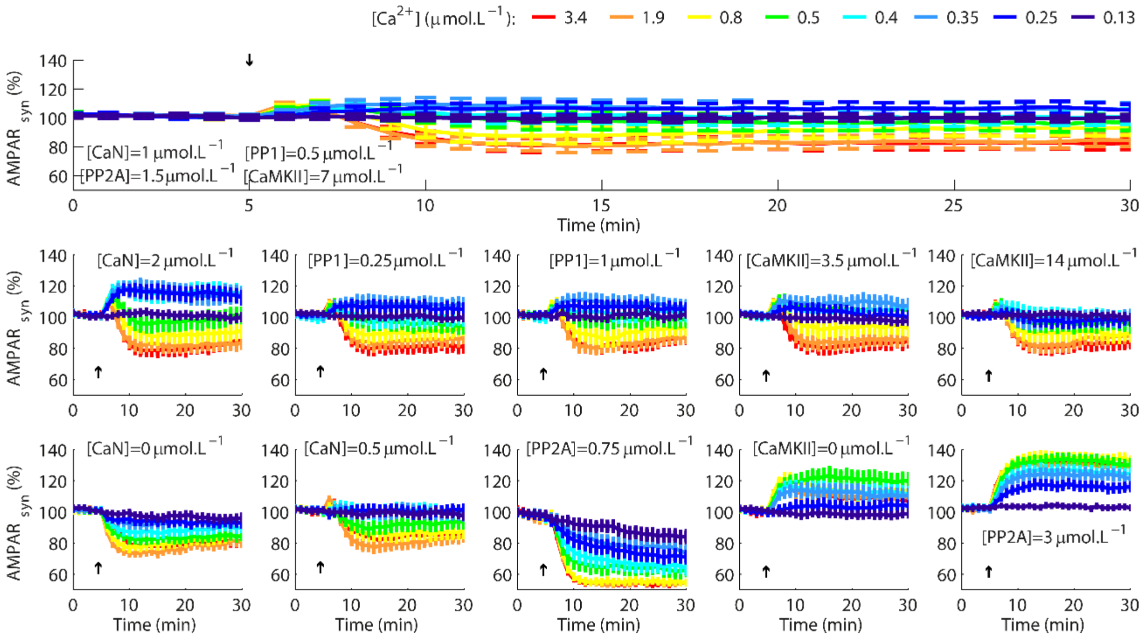

Supplementary Fig. S7. Time courses of synaptic plasticity for altered signalling networks. Time courses of $AMPAR_{syn}$ used to obtain the curves showed in Fig. 5. The first panel shows the results of the control model. The arrows indicated the moment of the occurrences of the Ca^{2+} pulses (duration of 20 s, the peak amplitudes are indicated in the legend). Each curve is the mean $\pm$ standard error of the mean calculated from 100 simulations.

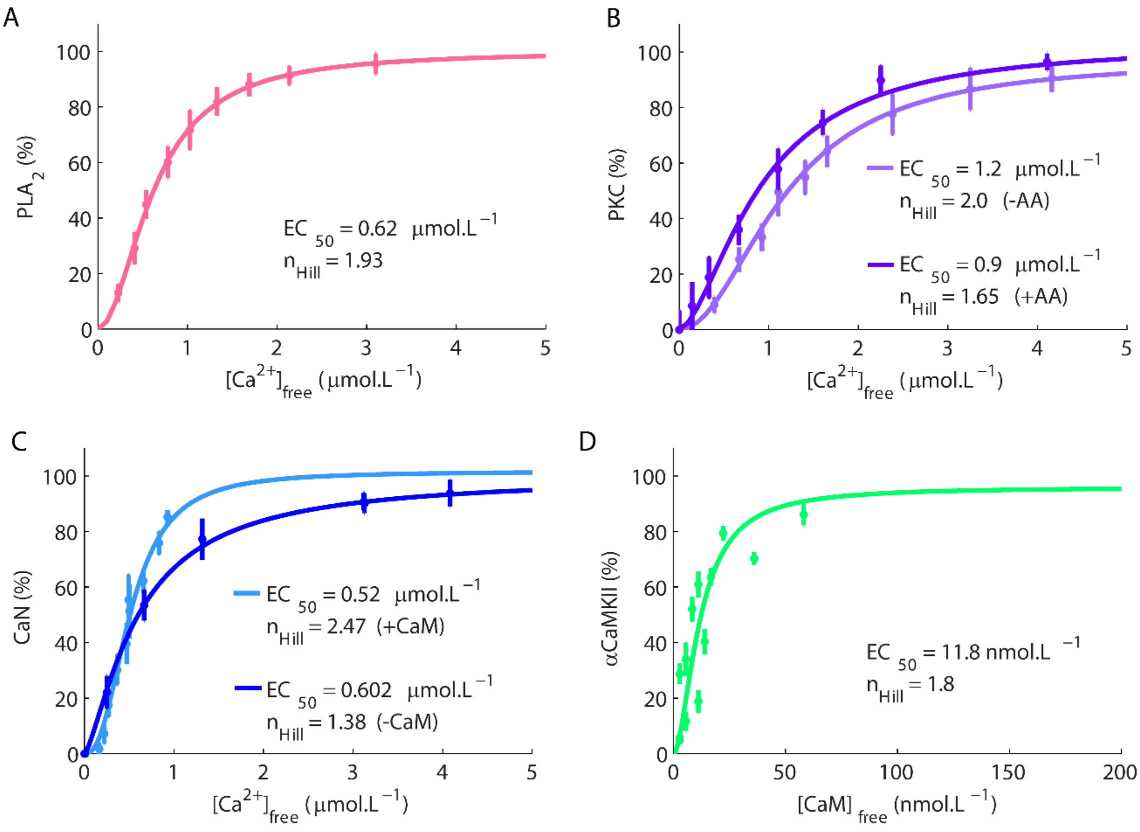

Supplementary Fig. S8. Dose-response curves. We used dose-response curves to validate the implementation of isolated Ca^{2+}-dependent components of the model through comparisons with experimental data. **(A)** Dose-response curve of the activation of PLA_2 as a function of [Ca^{2+}]. Experimental estimations reported n_{Hill} of 1.8[1] and EC_{50} of ~0.7 μmol.L^{-1} [2]. **(B)** Dose-response curves of PKC activation as a function of [Ca^{2+}] in the absence and in the presence of AA (10 μmol.L^{-1} of AA and 1 μmol.L^{-1} of PKC). Experimental studies reported EC_{50} of ~1.3 μmol.L^{-1} and n_{Hill} of 2.28 for the interaction of Ca^{2+} with PKC associated with membranes[3]. **(C)** Dose-response curves of CaN activation as a function of [Ca^{2+}] in the absence and in presence of CaM (10 μmol.L^{-1}). These results are consistent with published experimental observations, which reported EC_{50} of 0.67 μmol.L^{-1} and n_{Hill} of 1.2 for the interaction of CaN with Ca^{2+} in absence of CaM, and EC_{50} of approximately 0.5 μmol.L^{-1} and n_{Hill} around 2.5-3 in the presence of CaM [4,5]. **(D)** Dose-response curve of the autophosphorylation of αCaMKII as a function of [CaM] in presence of [Ca^{2+}] (50 μmol.L^{-1}). Experimental data reported n_{Hill} of 1.8-1.9 and EC_{50} around 4-20 nmol.L^{-1} [6–8] in the presence of nucleotides. Each dot is the average result of 10 simulations (mean ± standard error of the mean). The curves were fitted with 95% of confidence interval.

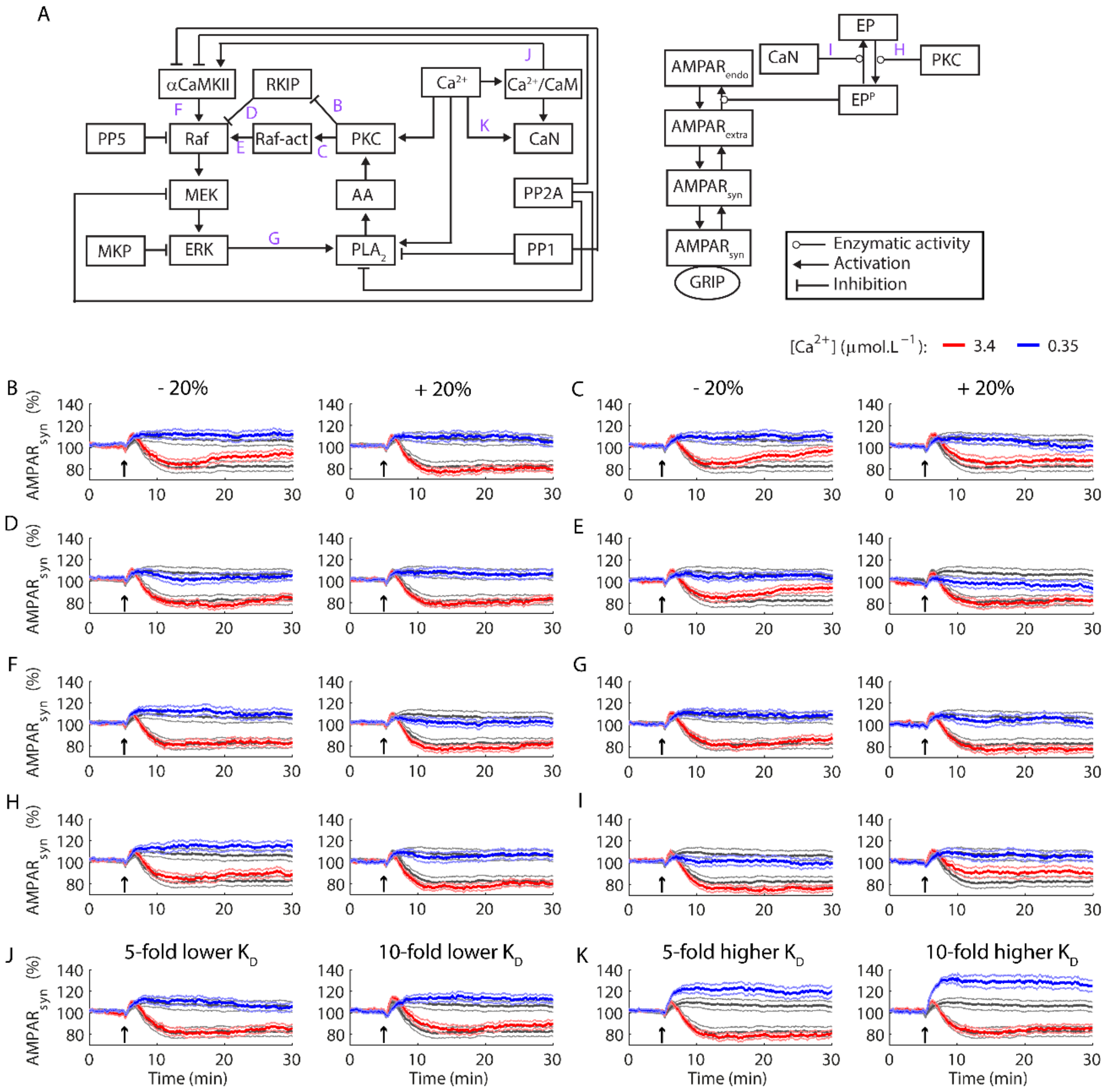

Supplementary Fig. S9. Sensitivity analyses. **(A)** Diagram of the model indicating the reactions analysed (purple letters B-I). **(B-I)** Simulations of the model with slower or faster rate constants (decrease of 20% and increase of 20%, respectively). For the analyses, we performed simulations of the model with a LTP and a LTD protocol and compared the results with the control model (gray lines, previously showed in Fig. 2B and Fig. S7). **(J-K)** Simulations of the model with changes in the affinities (indicated as dissociation constant, K_D) for the interaction of αCaMKII and Ca^{2+}/CaM **(J)** and Ca^{2+} with the subunit CNB of CaN **(K)**. Each curve is the average result of 50 runs of the model (mean ± standard error of the mean). Further details are described in the Supplementary Methods.

Supplementary Methods

The computational model presented in this study consists of a well-mixed compartment containing mechanisms of calcium ion (Ca^{2+}) dynamics and the components of the signalling network. The development of the model involved of two stages. Initially, we implemented each one of the Ca^{2+}-dependent components (calmodulin (CaM), calcineurin (CaN), protein kinase C (PKC), cytosolic phospholipases A_2 (PLA_2), and Ca^{2+}/Calmodulin protein kinase II α (αCaMKII)) isolated. We built these components based on the reaction mechanisms and parameters involved in their activations described in the literature. CaM and CaN were implemented according to previous descriptions[9,10]. The other components, including the members of the extracellular signal-regulated protein kinase (ERK) and the species involved with Ca^{2+}-dynamics, were implemented according to a previous model of cerebellar long-term depression (LTD)[11]. We validated the reactions and parameters used to simulate PKC, PLA_2, CaN, and αCaMKII by comparing their dose-response curves of activation with experimental data. After that, we coupled them with the other components of the model according to the diagrams showed in Fig. 1 and Supplementary Fig. S3.

The signalling network used to describe LTD and long-term potentiation (LTP) in the synapses between granule cells and Purkinje neurons consisted of a positive feedback loop formed by PKC-PLA_2-ERK pathway, which is implicated with LTD, and several protein phosphatases involved with LTP. The model of the positive feedback loop PKC-PLA_2-ERK pathway was based on a previous stochastic model[11] that we altered extensively to incorporate more recent experimental data. We included new components as part of the signalling machinery that simulates LTD (Raf kinase inhibitor protein (RKIP)[12], CaM, and αCaMKII[13]) and also made extensive changes in the reactions and parameters used to simulate PKC and PLA_2 based on experimental data. Therefore, the only components of the model that were simulated entirely using the same reactions and parameters implemented previously[11] were some members of ERK pathway, and the species (pumps and exchanger) involved with Ca^{2+}-dynamics as listed in Supplementary Table S1. Also, we changed the software used to build the model[11]. We implemented the model described in this work using BioNetGen[14], a rule-based software for modelling biochemical networks.

The signalling molecules implicated with LTP consisted of several phosphatases that were included in the previous model of LTD to counteract the activation of the components of the feedback loop. In addition to these phosphatases, we implemented

CaN, which is involved exclusively with LTP in Purkinje cells[15]. Moreover, we implemented an extensive mechanism of AMPA receptors (AMPARs) trafficking to simulate the expression of both LTP and LTD. The previous model of LTD simulated the trafficking of AMPARs and the persistent reduction in the population of synaptic AMPARs ($AMPAR_{syn}$) as the mechanism of LTD expression[11] based on several experimental evidences[16–21]. However, to simulate LTP, which is expressed as an increase in the population of $AMPAR_{syn}$[22], we expanded the mechanisms of AMPARs trafficking extensively to incorporate activity-driven mechanisms of exocytosis and reparametrized the reactions involved with endocytosis to balance the occurrence of both processes at rest and keep a stable basal population of $AMPAR_{syn}$. Most parameters of the model were obtained from the experimental literature. The other parameters were tuned manually to reproduce LTP and LTD in single synapses with magnitudes and time courses consistent with experimental data. All the other results presented in the work were emergent properties of the simulated system.

The details about the construction of the different components of the model are described in the following sections.

Ca^{2+} Dynamics

The Ca^{2+} dynamics of the model consisted of mechanisms responsible for the regulation of the intracellular Ca^{2+} concentration ([Ca^{2+}]), and comprised the Ca^{2+} influx and extrusion from the cytosol. The Ca^{2+} influx, used to elevate the levels of [Ca^{2+}] and promote plasticity, consisted of a first-order reaction that simulated pulses with specific durations and magnitudes. We also implemented a zero-order reaction that simulated a constant leak of Ca^{2+} to the cytosol to counteract the mechanisms of Ca^{2+} extrusion and sustain a basal [Ca^{2+}] of approximately 50 $nmol.L^{-1}$ [11]. We simulated the mechanisms of Ca^{2+} extrusion as described previously[11]. The extrusion was carried out by three species: the plasma membrane Ca^{2+}-ATPase (PMCA), the sarco/endoplasmic reticulum Ca^{2+}-ATPase (SERCA), and the sodium/Ca^{2+} exchanger (NCX). The description of the reactions and parameters of the Ca^{2+} dynamics are listed in Supplementary Table S1 (ID 1-4).

PLA_2

PLA_2 is a esterase composed by an α/β hydrolase domain and a C2 Ca^{2+}-binding domain that binds two Ca^{2+} with positive cooperativity[23,24]. The binding of Ca^{2+} to PLA_2

promotes its translocation to cellular membranes where its interfacial activation takes place and its substrates are located[23,24]. We simulated the association of two Ca^{2+} to PLA_2 in two successive and reversible steps. Each step consisted of a reaction of Ca^{2+} association and a reaction of Ca^{2+} dissociation ($Ca^{2+} + PLA_2 \underset{k_b}{\overset{k_f}{\rightleftharpoons}} \left(Ca^{2+}\right) PLA_2$, forward rate constant (k_f) = 1.83 µmol^{-1}.L.s^{-1} and backward rate constant (k_b) = 110 s^{-1}; for the second ion: $Ca^{2+} + \left(Ca^{2+}\right) PLA_2 \underset{k_b}{\overset{k_f}{\rightleftharpoons}} \left(Ca^{2+}\right)_2 PLA_2$ k_f = 11 µmol^{-1}.L.s^{-1}, k_b = 110 s^{-1})[1,23]. The binding of Ca^{2+} promoted the reversible translocation of PLA_2 to the membrane. We implemented the interaction of PLA_2 with the membrane as a pseudo-first order reaction (k_f = 26 s^{-1})[24]. Experimental estimations of the k_b for the dissociation reaction vary from 14 s^{-1} to 0.002 s^{-1} [24,25]. We set k_b as 0.05 s^{-1}. In the model, PLA_2 also interacted with the membrane in absence of Ca^{2+} or in presence of a single Ca^{2+} associated to its structure. For all these interactions, we kept k_f unchanged (26 s^{-1}). PLA_2 bound to a single Ca^{2+} dissociated from the membrane with k_b = 2.5 s^{-1}. In the absence of Ca^{2+}, we assumed a very weak affinity for the interaction of the membrane with PLA_2 and set k_b = 260 s^{-1}.

In the presence of the membrane, PLA_2 exhibits higher affinities for Ca^{2+} [23]. To simulate this process, we kept the k_f used for the interactions of PLA_2 with Ca^{2+} in absence of membrane unchanged, and recalculated the dissociation rate constants (k_b = 2.5 s^{-1} and 0.41 s^{-1} for the first and second ion, respectively)[1].

In the membrane, PLA_2 catalyses the release of arachidonic acid (AA). We implemented this catalysis according to a previous description[11]. The catalytic activity of PLA_2 is regulated by its phosphorylation catalyzed by ERK[25]. In the model, the phosphorylated PLA_2 catalyzed the released of AA with a higher catalytic rate (k_{cat})[2]. Additionally, the phosphorylation of PLA_2 increased its affinity for the membrane[25]. PLA_2 was dephosphorylated by protein phosphatase 2A (PP2A) and 1 (PP1)[11]. The reactions and parameters used to simulate PLA_2 are listed in Supplementary Table S1 (ID 5-22).

PKC

Several co-factors regulate PKC activity in a highly synergistic manner[3]. In the model, PKC interacted with Ca^{2+}, AA, and the cellular membrane with a random order. Typically, the association of PKC with Ca^{2+} and/or AA promotes its translocation to the cellular membrane, where its interfacial activation occurs[3,26]. In absence of $Ca^{2+}$and AA,

PKC interacts with the membrane with a weak affinity (K_D ~ 50 μmol.L^{-1})[27]. The association between PKC and the membrane, in the absence or presence of co-factors, was simulated as a pseudo-first order reaction, and the dissociation as a first-order reaction ($PKC \underset{k_b}{\overset{k_f}{\rightleftharpoons}} PKC_{memb}$, where PKC_{memb} states for PKC associated to the membrane). The k_f was set as 420 s^{-1} [28]. In the absence of Ca^{2+} and AA, we defined k_b = 8400 s^{-1} based on the weak affinity of this interaction[27]. In the presence of Ca^{2+} and AA, PKC has a stronger apparent affinity for the membrane[27], which we modelled through changes in the k_b for the dissociation reactions.

PKC interacts with three Ca^{2+} [29]. We simulated the binding/unbinding of the three ions as sequential and reversible reactions. One of the Ca^{2+}-binding site of PKC binds Ca^{2+} with higher affinity (K_D ~0.9 μmol.L^{-1}) than the other two Ca^{2+}-binding sites (K_D ~22.2 μmol.L^{-1})[29]. The values of k_f for the interaction of PKC with the first, second and third ion were 1111 μmol^{-1}.L.s^{-1}, 45.45 μmol^{-1}.L.s^{-1}, and 45.45 μmol^{-1}.L.s^{-1}, respectively. The values of k_b for the dissociation of PKC from the first, second and third ion was: 1000 s^{-1} [11,29]. PKC bound to the membrane has stronger affinities for Ca^{2+}. To simulate this property, we kept the rate constants of the binding of Ca^{2+} to PKC unchanged, and altered the rates of Ca^{2+} unbinding[11,28,29].

In the presence of Ca^{2+}, PKC has a stronger affinity for the membrane[27]. We simulated this property by changing the rate for PKC dissociation (k_b) from the membrane gradually according to the number of ions bound to the enzyme[11,24]. We kept k_f unchanged (420 s^{-1})[28].

PKC interacted with AA in the model with a stoichiometry of 1:1. The k_f for this interaction was 1 μmol^{-1}.L.s^{-1}, and k_b was 10 s^{-1} [11]. Typically, the activation of PKC by AA occurs in the presence of Ca^{2+} [26]. However, experimental evidences indicated that high concentrations of AA activates PKC in absence of Ca^{2+} [26]. We implemented the activation of PKC by AA assuming that it mediated its interaction with the membrane with a weak affinity, simulated with a k_b of 420 s^{-1}. The k_f for this interaction remained unchanged. The presence of AA in combination with a single Ca^{2+} associated to PKC promoted a reduction of the k_b for the dissociation of the enzyme from the membrane (k_b of 42 s^{-1}), without affecting the affinity of the enzyme for Ca^{2+} directly[30]. PKC bound to AA and two Ca^{2+} dissociated from the membrane with a k_b of 0.00017 s^{-1}. The complex PKC associated to AA and three Ca^{2+} had a k_b of 0.000017 s^{-1}, which implied that the dissociation of Ca^{2+} or AA preceded the unbinding of PKC from the membrane[24]. In the

model, PKC were catalytically active when it was associated to the membrane and three Ca^{2+}, to the membrane and AA, or to the membrane, AA and Ca^{2+}. The complete description of reactions and parameters used to simulate PKC are listed in Supplementary Table S1 (ID 23-37).

ERK pathway

ERK pathway consists of three protein kinases organized in a hierarchical sequence: Rapidly accelerated fibrossarcoma (Raf), mitogen-activated ERK kinase (MEK), and ERK. The activation of MEK and ERK require dual phosphorylations, which we simulated as distributive events[11]. However, based on recent experimental data, we implemented new mechanisms for the regulation of Raf.

We implemented Raf activation involving a single phosphorylation step[11]. In the feedback loop PKC-PLA_2-ERK, PKC participates in the activation of Raf, but indirectly[11]. Previously, the simulation of this process involved a Raf activator (Raf-act), a hypothetical kinase activated by PKC, that phosphorylated Raf in the model[11]. However, recent data revealed that PKC regulates Raf activation by phosphorylating RKIP[12], a Raf inhibitor. We then implemented the inhibition of Raf through its interaction with RKIP in the model, which prevents MEK activation. RKIP binds to Raf both in its phosphorylated and non-phosphorylated states[31]. Depending of the residues phosphorylated, the K_D for the interaction between Raf and RKIP vary from 0.1 to approximately 100 µmol.L^{-1} [31]. Nevertheless, as Raf has several putative phosphorylation residues[31], and many of them appear to regulate its affinity for RKIP[31], we opted to simulate the interaction between RKIP and Raf, phosphorylated and dephosphorylated, with the same affinity. The K_D for this interaction was 1 µmol.L^{-1} (k_f = 1 µmol^{-1}.L.s^{-1}, k_b = 1 s^{-1}). Thus, at rest, Raf was inhibited by RKIP in the model. The activation of PKC promoted the phosphorylation of RKIP[32] which resulted in a 100-fold reduction in its affinity for Raf in the model. The phosphorylation of RKIP by PKC was simulated with a K_M of 57 µmol.L^{-1} [32] that we converted in the velocity rate constants for the catalysis. We implemented RKIP dephosphorylation as a first-order process with a rate of 30 s^{-1} [33].

Though the disinhibition of Raf by PKC is involved in ERK regulation[12], this process alone was not enough to activate ERK pathway in the model because it did not cause Raf activation. As PKC, PLA_2, and ERK pathway integrate a positive feedback loop during LTD[34], they must present mechanisms that ensure the consecutive activation of all its components. Consequently, we decided to maintain the activation of Raf through

Raf-act implemented previously[11]. Therefore, PKC regulated Raf in the model through the inhibition of RKIP and the activation of Raf-act. Protein Phosphatase 5 (PP5) dephosphorylated Raf[35].

The activation of Raf promoted the activation of MEK and ERK, which we simulated as described previously[11]. ERK phosphorylates PLA_2 and contributes to sustain its activity in absence of Ca^{2+} [25]. This process was implemented as described previously[11], but we updated the rate constants used in the simulations[36]. The full description of the reactions and rate constants used to simulate ERK pathway is listed in Supplementary Table S1 (reactions 38-64).

CaM

To expand the LTD model to simulate LTP, we implemented CaM, a small Ca^{2+}-binding protein that acts both as a Ca^{2+} buffer and as a Ca^{2+} mediator in the activation of several proteins[37]. CaM is the only Ca^{2+} buffer implemented in the model. Purkinje cells contain high concentrations of several Ca^{2+} buffers that are important to shape the Ca^{2+} signals. However, we used Ca^{2+} pulses with controlled duration and amplitude in our model. Nevertheless, we implemented CaM to act as a Ca^{2+} mediator in the activation of CaN and αCaMKII. The model of the interaction of Ca^{2+} to CaM was implemented as described previously[9].

In brief, CaM is a globular protein containing two pairs of Ca^{2+} binding sites, one located on its N-terminal domain (sites I and II), and the other on its C-terminal domain (sites III and VI). Ca^{2+} binds to the pair of Ca^{2+}-binding sites of each CaM domain sequentially and with positive cooperativity[9]. There is no interdomain cooperativity[9]. We assumed that each CaM domain has two macroscopic association constants, K_1 and K_2. K_1 was defined as the sum of the microscopic equilibrium constants. Thus, for each pair of Ca^{2+}-binding sites of CaM, K_1 is given as follows:

$$K_{N1} = k_I + k_{II} \text{, for the N-terminal domain} \quad (1)$$

$$K_{C1} = k_{III} + k_{IV} \text{, for the C-terminal domain} \quad (2)$$

where k_I, k_{II}, k_{III}, and k_{IV}, correspond to the microscopic equilibrium constants of the Ca^{2+}-binding sites I, II, III, and IV, respectively. K_2 is given by the cooperativity for the binding of a second Ca^{2+} to either CaM globular domain as follows:

$$K_{N2} = k_n k_I k_{II} \text{, for the N-terminal domain} \tag{3}$$

$$K_{C2} = k_c k_{III} k_{IV} \text{, for the C-terminal domain} \tag{4}$$

k_n and k_c are the intradomain cooperative constants for the binding of the second Ca^{2+} to the N- and C-terminal domains. A full description of the reactions and references used in the model of CaM was published previously[9]. The parameters and reactions implemented to simulate the interaction between Ca^{2+} and CaM are listed in Supplementary Table S1 (ID 65-72).

CaN

CaN is a heterodimer formed by a catalytic subunit (CNA), which contains a Ca^{2+}/CaM-binding site, and a regulatory subunit (CNB) with four Ca^{2+}-binding sites[4,38]. The interaction of Ca^{2+} with CNB is necessary for binding of Ca^{2+}/CaM to CNA[4,39].

CNB contains two globular domains, each one with a pair of Ca^{2+}-binding sites. CNB is structurally similar to CaM and troponin C, in consequence, we simulated the binding/unbinding of Ca^{2+} to the four Ca^{2+}-binding sites of CNB as having intradomain cooperativity, but without interdomain cooperativity. To estimate the microscopic parameters for the interaction of Ca^{2+} to CNB, we used the same approach applied to CaM (equations 1-4). The macroscopic binding constants for Ca^{2+}-binding sites of CNB are 0.094 $\mu mol.L^{-1}$ (K_{C1}), 0.036 $\mu mol.L^{-1}$ (K_{C2}), 1.1 $\mu mol.L^{-1}$ (K_{N1}), and 0.6 $\mu mol.L^{-1}$ (K_{N2})[40]. We assumed that the microscopic affinity for both sites of a given CaN domain are equivalent, and calculated the values of k_{III} and k_{IV} (0.047 $\mu mol.L^{-1}$), k_I and k_{II} (0.55 $\mu mol.L^{-1}$), and the values of the cooperative constant for the C-terminal (k_c = 16.29 $\mu mol^{-1}.L$), and N-terminal (k_n = 2 $\mu mol^{-1}.L$). The k_b for the dissociation of the first Ca^{2+} from the C-terminal was defined as 0.03 s^{-1} [38], which resulted in a k_f of 0.64 $\mu mol^{-1}.L.s^{-1}$. We used equal values of k_f for the binding of Ca^{2+} to both sites on the same terminal domain. We calculated the k_b for the dissociation of Ca^{2+} from the second site occupied in the C-terminal as 0.0018 s^{-1}. For the sites of the N-terminal, we used a k_b of 0.05 s^{-1} [38] for the

first Ca^{2+}, and calculated the k_f (0.09 $\mu mol^{-1}L.s^{-1}$). We used the same k_f for the binding of the second ion. The k_b for the unbinding of the second Ca^{2+} was 0.025 s^{-1}.

CNA interacted with Ca^{2+}/CaM after the binding of Ca^{2+} to CNB. CaN binds to Ca^{2+}/CaM fully or partially loaded with Ca^{2+} [41]. We simulated the interaction of CaN with fully loaded Ca^{2+}/CaM using a k_f of 46 $\mu mol^{-1}.L.s^{-1}$ and a k_b of 0.0012 s^{-1} [42]. The interaction between CaM and its targets occurs through hydrophobic residues exposed after its binding to Ca^{2+}. The association of a single Ca^{2+} can shift CaM conformation from closed to open and exposes residues for target interaction[43], though the binding of a pair of ions is important for stabilizing the open conformation of each domain[43]. Because the binding of a single ion can change CaM conformation, we assumed that CaN interact with CaM bound to three or two Ca^{2+} with the same affinity reported for the interaction with CaM fully saturated. However, at least one Ca^{2+}-binding site of each CaM domain had to be filled. For interactions of CaN with CaM with Ca^{2+} in only one of its domains, we kept the k_f (46 $\mu mol^{-1}.L.s^{-1}$) unchanged. Then, we recalculated the k_b using affinities for the association of CaN with isolated CaM domains (K_D = 1 for the C-terminal (k_b = 46 s^{-1}), and K_D = 7 $\mu mol.L^{-1}$ for the N-terminal (k_b = 322 s^{-1}))[41].

Similarly to other CaM targets[37], CaN increases the affinity of Ca^{2+} for CaM[4,41]. This process was implemented using equations (1)-(4). The presence of CaM targets usually alters the rate constants of Ca^{2+} dissociation from CaM structure [44]. Based on this fact, the k_bs for the dissociation of the first and second ion to each CaM domain associated to CaN were calculated considering the microscopic binding for the sites k_I and k_{II} (0.8 $\mu mol^{-1}.L$) and k_{III} and k_{IV} (5 $\mu mol^{-1}.L$). We kept the cooperative constants k_n (80 $\mu mol^{-1}.L$), and k_c (200 $\mu mol^{-1}.L$) unchanged by the presence of CaN [9]. The complete description of the reactions and parameters used to simulate CaN was published previously[10] and is listed in Supplementary Table S1 (reactions 73-87).

αCaMKII

αCaMKII is a multimeric holoenzyme composed of 12-14 subunits, organized as an assembly of 'vertical dimers'[45]. Each subunit consists of an N-terminal kinase domain, a regulatory domain, a linker, and a C-terminal association domain[45]. The regulatory domain docks to the substrate-recognition site of the kinase domain and retains the subunit in an inactive state[45]. The binding of Ca^{2+}/CaM to the regulatory domain activates αCaMKII kinase activity. Each αCaMKII subunit binds to one Ca^{2+}/CaM (*23*)[45]. αCaMKII interacts with CaM fully or partially saturated with Ca^{2+} [46,47]. The association

of Ca^{2+}/CaM promotes the trans-autophosphorylation of the residue Thr^{286}, which requires the kinase domain of one subunit to phosphorylate the kinase domain of another subunit within the same holoenzyme[45]. The autophosphorylation of αCaMKII maintains it in a partial Ca^{2+}-independent activated state, which is further stimulated by Ca^{2+}/CaM[48].

The multimeric structure of αCaMKII with multiple different states makes it a difficult enzyme to model. In consequence, the majority of the available models of αCaMKII adopted strategies of simplification, which typically consist of reducing the states considered for each subunit and reducing the size of the holoenzyme[49,50]. In our model, we reduced the size of the holoenzyme to two subunits implemented with a detailed mechanism of interaction with Ca^{2+}/CaM. We simulated two subunits instead of one because αCaMKII binds Ca^{2+}/CaM with positive cooperativity[6,45].

The interaction of the first subunit with Ca^{2+}/CaM was simulated with a k_f of 20 $\mu mol^{-1}.L.s^{-1}$ [7,8] and k_b of 20 s^{-1}. For the binding of the second Ca^{2+}/CaM, we implemented a reduction in the k_b (18 s^{-1}) to simulate the cooperativity for its interaction with αCaMKII. We manually tuned this value to fit the hill coefficient (n_{hill}) reported for αCaMKII interaction with Ca^{2+}/CaM (~1.9)[6].

Experimental findings reported that αCaMKII interacts with CaM partially loaded with Ca^{2+} [46,47]. We assumed that αCaMKII interacts with CaM with at least one Ca^{2+} bound to each one of its globular domains with the same rate constants used for the interaction of αCaMKII with CaM fully saturated with Ca^{2+}. The association of αCaMKII with CaM with Ca^{2+} bound only to its N-terminal domain occurs with a very weak affinity[46,47]. Consequently, we omitted this interaction in the model for simplicity. The interaction between αCaMKII and CaM with Ca^{2+} bound only to the Ca^{2+}-binding sites of the C-terminal domain was implemented considering an affinity 250-fold lower than the affinity used for the interaction between αCaMKII and CaM fully loaded with Ca^{2+}. Experimental reports indicated changes in this affinity ranging from ~100-500-fold [46,47]. We simulated this reduction in the affinity by changing the k_b for the dissociation of CaM from αCaMKII (k_b = 5000 s^{-1} and 4500 s^{-1} for the first and second Ca^{2+}/CaM molecule, respectively).

The presence of αCaMKII increases the affinity between CaM and Ca^{2+}. However, αCaMKII changes the affinity of the Ca^{2+}-binding sites of CaM for Ca^{2+} asymmetrically[44,47]. We simulated this process using a reduction of the k_bs for the dissociation of Ca^{2+} from CaM bound to αCaMKII of 100-fold and 20-fold for the Ca^{2+}-binding sites of the N and C-terminal domains, respectively[44,47]. The autophosphorylation

of αCaMKII did not promote a further enhancement in the affinity between CaM and Ca^{2+} in the model.

Although αCaMKII can interact with CaM partially saturated with Ca^{2+}, the filling of the Ca^{2+} bindings sites I, III and IV of CaM are necessary for αCaMKII activation[51]. For simplicity, we assumed that only αCaMKII associated to two molecules of CaM fully saturated with Ca^{2+} could undergo its trans-autophosphorylation. Two molecules of CaM were necessary because αCaMKII autophosphorylation is an inter-subunit process[45]. We implemented the autophosphorylation of αCaMKII as a first-order reaction with a rate constant of 5 s^{-1} [51]. After its autophosphorylation, αCaMKII has a significant increase in its affinity for CaM[52]. We used a 1000-fold reduction in the k_bs for the dissociation of CaM from αCaMKII to implement this change in affinity[52].

We estimated the number of copies of our dimeric αCaMKII in 250[53], which corresponds to 500 subunits.

Classically, it was assumed that the autonomy of αCaMKII persisted for hours to sustain long-term forms of synaptic plasticity, especially hippocampal LTP. However, recent experimental findings reported that, in dendritic spines, during hippocampal LTP, the activation of αCaMKII persists only for seconds[54]. Based on these findings, in our model, autonomous αCaMKII was rapidly inactivated by dephosphorylation after its activation caused by a brief Ca^{2+} pulse. Two enzymes, PP1 and PP2A, dephosphorylate αCaMKII[55]. In hippocampal dendritic spines, evidences indicated that PP1 is likely to play a major role in αCaMKII dephosphorylation than PP2A in the postsynaptic density (PSD)[55]. Outside the PSD, PP2A is the main responsible for αCaMKII dephosphorylation[55]. Nevertheless, in Purkinje cells, these differences between PP1 and PP2A activity on αCaMKII have not been investigated. Thus, in the model, both PP1 and PP2A dephosphorylated αCaMKII with equivalent rate constants estimated from experimental reports[55,56].

In the model, αCaMKII acted as a Raf kinase[57,58]. Most models of CaMKII assumed that its autonomous activity is equal to its activity in the presence of Ca^{2+}/CaM. However, the catalytic activity of autonomous αCaMKII toward its substrates is lower (15-25 %)[48]. To simulate the phosphorylation of Raf by αCaMKII, we assumed that only αCaMKII associated to two molecules of fully saturated Ca^{2+}/CaM had maximum catalytic activity. Autonomous αCaMKII or αCaMKII bound to all the other states of Ca^{2+}/CaM where simulated having a catalytic rate constant (k_{cat}) that corresponded to

20% of its maximum activity. The description of the reactions and parameters used to simulate αCaMKII is listed in Supplementary Table S1 (reactions 88-108).

AMPAR trafficking

We simulated AMPARs trafficking using first-order reactions for the reversible lateral diffusion of the synaptic receptors ($AMPAR_{syn}$) to extra-synaptic regions ($AMPAR_{extra}$)[11]. We estimated the rate constants for these reactions using the diffusion coefficients of AMPARs and the length of the synaptic and extra-synaptic areas[11,59,60]. The endocytosis of AMPARs occurred from extra synaptic sites and required the catalytic activity of a phosphorylated endocytic protein (EP). The activation of PKC during LTD caused the synaptic depression through the phosphorylation of $AMPAR_{syn}$[11]. PP2A counteracted PKC activity[11]. The phosphorylation of AMPARs by PKC did not alter their lateral diffusion, but increased their endocytosis rate. We implemented the exocytosis of internalized AMPARs ($AMPAR_{endo}$) as a first-order reaction that inserted the receptors in the extra-synaptic regions with an exocytosis rate (0.006 s^{-1}) tuned to obtain time courses of LTP observed in Purkinje neurons. The exocytosis of AMPAR required their dephosphorylation in the model.

Part of the $AMPAR_{syn}$ interacted with the glutamate-receptor interacting protein (GRIP) and were simulated as immobile[16,60]. The phosphorylation of $AMPAR_{syn}$ promoted a decrease in their affinity for GRIP and reduced the amount of receptors that interacted with it[16]. We used a K_D of 1 µmol.L^{-1} for the binding/unbinding of GRIP and $AMPAR_{syn}$. These rates were based on experimental and theoretical data[60]. For the interaction of the phosphorylated $AMPAR_{syn}$ with GRIP, we kept k_f unchanged and increased k_b by seven-fold[16,60]. As a result of this change of k_b, a larger pool of $AMPAR_{syn}$ could undergo lateral diffusion and endocytosis, which promoted LTD in the model.

To simulate LTP, we assumed that the internalization of AMPARs required the catalytic activity of phosphorylated EP. This assumption was based on recent data that demonstrated that phosphorylated syndapin participates in the endocytosis of AMPARs through associations with protein interacting with C-kinase 1 (PICK1), and, possibly, dynamin[19]. Several proteins involved with vesicle endocytosis are phosphorylated by PKC and dephosphorylated by CaN[61,62]. Moreover, both PKC and CaN are directly involved with AMPARs trafficking[16–19,63]. Thus, in our model, CaN counteracted the action of PKC on EP. The dephosphorylation of EP blocked the internalization of AMPARs without affecting their constitutive exocytosis. To define the rate constants for

these reactions, we used a higher K_m for PKC (5 µmol.L^{-1} for PKC and 11 µmol.L^{-1} for CaN), but higher rate constants for CaN. This assumption was important to favour the fast action of CaN on EP because CaN has no sustained activity in our model. We implemented PKC action with a higher K_m than CaN to guarantee the phosphorylation of EP under basal conditions to sustain the constitutive AMPARs trafficking. The full description of the reactions and parameters used to simulate AMPARs trafficking is listed in Supplementary Table S1 (reactions 109-129).

Additional methods

For the molecules directly activated by Ca^{2+}, we fitted dose-response curves of activation and compared them with published experimental data for validation. Each molecule was analysed isolated from the other components of the model. We performed these analyses for PLA_2, PKC, CaN and αCaMKII. We validated CaM previously[9]. The other components were validated as part of the LTD model published before[11].

For each dose-response curve, we varied systematically the initial [Ca^{2+}] and, after the system has reached steady-state, we verified the remaining free [Ca^{2+}] ($[Ca^{2+}]_{free}$) and the concentration of the active enzyme under analysis. Then, we fitted the dose-response curves with the equation:

$$[P] = [P_{\max}]\frac{[Ca^{2+}]^{n_{Hill}}}{EC_{50}^{n_{Hill}} + [Ca^{2+}]^{n_{Hill}}} \tag{5}$$

where *[P]* is the percentage of the activate enzyme, $[P]_{max}$ is its percentage of maximum activation, n_{Hill} is the Hill coefficient, and EC_{50} is the [Ca^{2+}] required to activate the half maximum amount of *[P]*. The parameters of the dose-response curves, n_{Hill} and EC_{50}, were compared with experimental data.

The parameters obtained for the dose-response curve of the activation of PLA_2 as a function of [Ca^{2+}] (Supplementary Fig. S8A) are in accordance with experimental data, which reported an apparent K_{Ca} (EC_{50}) of ~0.7 µmol.L^{-1} [2]. The n_{Hill} for the interaction of Ca^{2+} with PLA_2 associated with membranes was estimated in 1.8[1]. We also obtained parameters for the activation of PKC consistent with experimental findings, which reported EC_{50} of ~1.3 µmol.L^{-1} and n_{Hill} of 2.28 for the interaction of Ca^{2+} with PKC associated with membranes[3] (Supplementary Fig. S8B). PKC is known to be activated by combinations of its co-factors in a synergistic manner[3]. Thus, in the presence of a constant

concentration of AA (10 μmol.L^{-1}), we verified a decrease in the EC_{50} for the activation of PKC by Ca^{2+} and a slight reduction in its n_{Hill} (Supplementary Fig. S8B). We validated the reactions and parameters used to simulate CaN by verifying its Ca^{2+} requirement in the absence and in the presence of saturating CaM (1 μmol.L^{-1} of CaN and 10 μmol.L^{-1} of CaM, respectively). The parameters of the dose-response curves (Supplementary Fig. S8C) obtained by fitting equation (5) are consistent with published experimental observations, which reported EC_{50} of 0.67 μmol.L^{-1} and n_{Hill} of 1.2 for the interaction of CaN to Ca^{2+} in absence of saturating CaM, and EC_{50} of approximately 0.5 μmol.L^{-1} and n_{Hill} around 2.5-3 in the presence of saturating CaM[4,5]. Note that the model of CaN used in this work was published previously[10]. To validate the model of αCaMKII activation, we verified its autophosphorylation as a function of CaM concentration (in equation (5), we replaced [Ca^{2+}] with [CaM]) in the presence of Ca^{2+} (1 μmol.L^{-1} of αCaMKII, 50 μmol.L^{-1} of Ca^{2+}) (Supplementary Fig. S8D). Experimental data reported n_{Hill} of 1.8-1.9 and K_D around 5-20 nmol.L^{-1} [6–8] in the presence of nucleotides (ATP and ADP), which we used for comparison with EC_{50}. Note that, in the absence of nucleotides, αCaMKII interacts with CaM with a 10-fold weaker affinity[6–8]. However, ATP and ADP, which are co-substrate and co-product of αCaMKII, respectively, are present in concentrations well beyond the levels required to saturate αCaMKII in the cells.

Sensitivity Analyses

Most parameters used in the model were determined experimentally. However, some parameters have not been determined experimentally and were tuned to reproduce the curves of synaptic plasticity. We performed sensitivity analyses to verify which one of these parameters produce the most variation in the time courses of opposite forms of synaptic plasticity. However, we restricted the sensitivity analyses to some reactions of the model because many of its components were analysed previously[11]. The analyses focused on the rate constants used for the reactions involved in the connections between PKC and ERK pathway through the activation of Raf, an important bottleneck for the activation of the positive feedback loop involved with LTD. We also analysed the rate constants used for the phosphorylation of PLA_2 by ERK and for the phosphorylation/dephosphorylation of EP by PKC and CaN. Supplementary Fig. S9A indicated all the reactions analysed. The analyses consisted in systematic changes in the rate constants (increase of 20% or decrease of 20%) used in the connections between the selected components. All the reactions involved in each connection point analysed were

altered simultaneously and by the same factor. So, for instance, for a catalytic reaction involving an enzyme E, a substrate S and the formation of a product P ($E + S \underset{k_b}{\overset{k_f}{\rightleftharpoons}} ES \xrightarrow{k_{cat}} E + P$), we altered k_f, k_b, and k_{cat} simultaneously (increase of 20% or decrease of 20%) for the analysis. Then, we performed simulations and compared the results with the control model (Supplementary Fig. S9B-I, gray lines) during the occurrence of LTP and LTD in a population of synapses. The results obtained confirmed that the mechanisms involved in the activation of Raf are important bottlenecks of the model of LTD (Supplementary Fig. S9B-F). As a result, reductions in the rate constants for the phosphorylation of RKIP and Raf-act by PKC affected the stability of LTD, but did not alter LTP (Supplementary Fig. S9B-C). Changes in the rate constants for the inhibition of Raf by RKIP promoted no significant modifications in the results of the model (Supplementary Fig. S9D). The occurrence of both LTP and LTD were sensitive to changes in the rate constants for the activation of Raf by Raf-act (Supplementary Fig. S9E), but only slightly affected by alterations in the activation of Raf by αCaMKII (Supplementary Fig. S9F). Changes in the parameters for the phosphorylation of PLA_2 by ERK promoted no modifications of LTP and LTD (Supplementary Fig. S9G). The reduction of the rate constants for the phosphorylation of EP by PKC increased the magnitude of LTP without affecting LTD. However, the increase of the same rate constants had no effects in the model (Supplementary Fig. S9H). In contrast, the decrease of the rate constants of EP dephosphorylation catalyzed by CaN altered the magnitudes of LTP and LTD, but the increase of the same rate constants affected only LTD (Supplementary Fig. S9I). In addition to this analysis, we verified the impacts of the affinity for the interaction between Ca^{2+}/CaM and αCaMKII and the affinities of CaN for binding to Ca^{2+} in the results obtained. As mentioned previously, in presence of nucleotides, αCaMKII has an affinity for Ca^{2+}/CaM that is approximately 10-fold higher than the affinity measured in absence of nucleotides[6–8]. In the cells, nucleotides are present in concentrations high above what is required to saturate αCaMKII. However, as many works estimated the affinity for the interaction of Ca^{2+}/CaM and αCaMKII in absence of nucleotides, we decreased the affinity for the binding of Ca^{2+}/CaM to αCaMKII by 5- and 10-fold to verify its impact on the model (Supplementary Fig. S9J). None of these changes resulted in a significant change in the results of the model (Supplementary Fig. S9J), which indicated that the affinity for the interaction of Ca^{2+}/CaM and αCaMKII used in the model was not crucial for the results observed. Next,

we accessed the effects of the affinities for the interactions of Ca^{2+} with the Ca^{2+}-binding sites of the subunit CNB of CaN on the model. As described previously, the binding of Ca^{2+} to the Ca^{2+}-bindings sites of CNB is a precondition for the high affinity[42] interaction of Ca^{2+}/CaM with CNA[4,38,39]. Moreover, though CNA interacts with Ca^{2+}/CaM with a very high affinity, the overall Ca^{2+} requirement of CaN to become activated is in the range of 0.5-0.7 $\mu mol.L^{-1}$ in the model, which is fully consistent with the experimental literature[4,5,38]. However, to verify whether the Ca^{2+} requirement of CaN could act as a bottleneck to the results observed in this work, we enhanced the affinities for the bindings of Ca^{2+} to the Ca^{2+}-bindings sites of CNB by 5-fold and 10-fold (Supplementary Fig. S9K). The results of this manipulation demonstrated that the increase in the affinity of Ca^{2+} for CNB increased the magnitude of LTP occurrences without affecting LTD (Supplementary Fig. S9K). In the model, CaN is involved only with LTP, which is consistent with previous studies[15,64]. LTD requires the activation of a positive feedback loop, which presents a robust and sustained activity that occludes the activation of CaN and the expression of LTP. As CaN does not influence the activation of the feedback loop, increasing its activation by changing its affinity for Ca^{2+} does not alter the occurrence of LTD. Therefore, favouring the activation of CaN by increasing its affinity for Ca^{2+} promoted only an enhancement in the magnitude of LTP. Thus, the data presented in our work are not based on the affinity of Ca^{2+}/CaM for αCaMKII or on the affinity of Ca^{2+} for CaN, but resulted from the whole system simulated.

References

1. Nalefski, E. A. & Falke, J. J. Cation charge and size selectivity of the C2 domain of cytosolic phospholipase A(2). *Biochemistry (Mosc.)* **41,** 1109–22 (2002).
2. Tucker, D. E. *et al.* Role of phosphorylation and basic residues in the catalytic domain of cytosolic phospholipase A2alpha in regulating interfacial kinetics and binding and cellular function. *J Biol Chem* **284,** 9596–611 (2009).
3. Egea-Jiménez, A. L., Pérez-Lara, A., Corbalán-García, S. & Gómez-Fernández, J. C. Phosphatidylinositol 4,5-bisphosphate decreases the concentration of Ca2+, phosphatidylserine and diacylglycerol required for protein kinase C α to reach maximum activity. *PLoS One* **8,** e69041 (2013).
4. Stemmer, P. M. & Klee, C. B. Dual calcium ion regulation of calcineurin by calmodulin and calcineurin B. *Biochemistry (Mosc.)* **33,** 6859–66 (1994).

5. Feng, B. & Stemmer, P. M. Ca2+ binding site 2 in calcineurin-B modulates calmodulin-dependent calcineurin phosphatase activity. *Biochemistry (Mosc.)* **40,** 8808–14 (2001).
6. Forest, A. *et al.* Role of the N- and C-lobes of calmodulin in the activation of Ca(2+)/calmodulin-dependent protein kinase II. *Biochemistry (Mosc.)* **47,** 10587–99 (2008).
7. Tzortzopoulos, A. & Török, K. Mechanism of the T286A-mutant alphaCaMKII interactions with Ca2+/calmodulin and ATP. *Biochemistry (Mosc.)* **43,** 6404–14 (2004).
8. Török, K., Tzortzopoulos, A., Grabarek, Z., Best, S. L. & Thorogate, R. Dual effect of ATP in the activation mechanism of brain Ca(2+)/calmodulin-dependent protein kinase II by Ca(2+)/calmodulin. *Biochemistry (Mosc.)* **40,** 14878–90 (2001).
9. Antunes, G., Sebastião, A. M. & Simoes de Souza, F. M. Mechanisms of Regulation of Olfactory Transduction and Adaptation in the Olfactory Cilium. *PLoS One* **9,** e105531 (2014).
10. Antunes, G., Roque, A. C. & Simoes de Souza, F. M. Modelling intracellular competition for calcium: kinetic and thermodynamic control of different molecular modes of signal decoding. *Sci. Rep.* **6,** 23730 (2016).
11. Antunes, G. & De Schutter, E. A stochastic signaling network mediates the probabilistic induction of cerebellar long-term depression. *J Neurosci* **32,** 9288–300 (2012).
12. Yamamoto, Y. *et al.* Raf kinase inhibitory protein is required for cerebellar long-term synaptic depression by mediating PKC-dependent MAPK activation. *J Neurosci* **32,** 14254–64 (2012).
13. Hansel, C. *et al.* alphaCaMKII Is essential for cerebellar LTD and motor learning. *Neuron* **51,** 835–43 (2006).
14. Faeder, J. R., Blinov, M. L. & Hlavacek, W. S. Rule-based modeling of biochemical systems with BioNetGen. *Methods Mol Biol* **500,** 113–67 (2009).
15. Schonewille, M. *et al.* Purkinje cell-specific knockout of the protein phosphatase PP2B impairs potentiation and cerebellar motor learning. *Neuron* **67,** 618–28 (2010).
16. Matsuda, S., Launey, T., Mikawa, S. & Hirai, H. Disruption of AMPA receptor GluR2 clusters following long-term depression induction in cerebellar Purkinje neurons. *EMBO J* **19,** 2765–74 (2000).

17. Matsuda, S., Mikawa, S. & Hirai, H. Phosphorylation of serine-880 in GluR2 by protein kinase C prevents its C terminus from binding with glutamate receptor-interacting protein. *J Neurochem* **73,** 1765–8 (1999).
18. Chung, H. J., Steinberg, J. P., Huganir, R. L. & Linden, D. J. Requirement of AMPA receptor GluR2 phosphorylation for cerebellar long-term depression. *Science* **300,** 1751–5 (2003).
19. Anggono, V. *et al.* PICK1 interacts with PACSIN to regulate AMPA receptor internalization and cerebellar long-term depression. *Proc Natl Acad Sci U A* **110,** 13976–81 (2013).
20. Linden, D. J. The expression of cerebellar LTD in culture is not associated with changes in AMPA-receptor kinetics, agonist affinity, or unitary conductance. *Proc Natl Acad Sci U A* **98,** 14066–71 (2001).
21. Wang, Y. T. & Linden, D. J. Expression of cerebellar long-term depression requires postsynaptic clathrin-mediated endocytosis. *Neuron* **25,** 635–47 (2000).
22. Kakegawa, W. & Yuzaki, M. A mechanism underlying AMPA receptor trafficking during cerebellar long-term potentiation. *Proc Natl Acad Sci U A* **102,** 17846–51 (2005).
23. Nalefski, E. A., Slazas, M. M. & Falke, J. J. Ca2+-signaling cycle of a membrane-docking C2 domain. *Biochemistry (Mosc.)* **36,** 12011–8 (1997).
24. Nalefski, E. A. *et al.* C2 domains from different Ca2+ signaling pathways display functional and mechanistic diversity. *Biochemistry (Mosc.)* **40,** 3089–100 (2001).
25. Das, S., Rafter, J. D., Kim, K. P., Gygi, S. P. & Cho, W. Mechanism of group IVA cytosolic phospholipase A(2) activation by phosphorylation. *J Biol Chem* **278,** 41431–42 (2003).
26. O'Flaherty, J. T., Chadwell, B. A., Kearns, M. W., Sergeant, S. & Daniel, L. W. Protein kinases C translocation responses to low concentrations of arachidonic acid. *J Biol Chem* **276,** 24743–50 (2001).
27. Sánchez-Bautista, S., Marín-Vicente, C., Gómez-Fernández, J. C. & Corbalán-García, S. The C2 domain of PKCalpha is a Ca2+ -dependent PtdIns(4,5)P2 sensing domain: a new insight into an old pathway. *J Mol Biol* **362,** 901–14 (2006).
28. Kohout, S. C., Corbalán-García, S., Torrecillas, A., Goméz-Fernandéz, J. C. & Falke, J. J. C2 domains of protein kinase C isoforms alpha, beta, and gamma: activation parameters and calcium stoichiometries of the membrane-bound state. *Biochemistry (Mosc.)* **41,** 11411–24 (2002).

29. Torrecillas, A., Laynez, J., Menéndez, M., Corbalán-García, S. & Gómez-Fernández, J. C. Calorimetric study of the interaction of the C2 domains of classical protein kinase C isoenzymes with Ca2+ and phospholipids. *Biochemistry (Mosc.)* **43,** 11727–39 (2004).
30. López-Nicolás, R., López-Andreo, M. J., Marín-Vicente, C., Gómez-Fernández, J. C. & Corbalán-García, S. Molecular mechanisms of PKCalpha localization and activation by arachidonic acid. The C2 domain also plays a role. *J Mol Biol* **357,** 1105–20 (2006).
31. Park, S. *et al.* Regulation of RKIP binding to the N-region of the Raf-1 kinase. *FEBS Lett* **580,** 6405–12 (2006).
32. Granovsky, A. E. *et al.* Raf kinase inhibitory protein function is regulated via a flexible pocket and novel phosphorylation-dependent mechanism. *Mol Cell Biol* **29,** 1306–20 (2009).
33. Shin, S. Y. *et al.* Functional roles of multiple feedback loops in extracellular signal-regulated kinase and Wnt signaling pathways that regulate epithelial-mesenchymal transition. *Cancer Res* **70,** 6715–24 (2010).
34. Tanaka, K. & Augustine, G. J. A positive feedback signal transduction loop determines timing of cerebellar long-term depression. *Neuron* **59,** 608–20 (2008).
35. Dhillon, A. S., von Kriegsheim, A., Grindlay, J. & Kolch, W. Phosphatase and feedback regulation of Raf-1 signaling. *Cell Cycle* **6,** 3–7 (2007).
36. Roskoski, R. ERK1/2 MAP kinases: Structure, function, and regulation. *Pharmacol. Res.* **66,** 105–143 (2012).
37. Xia, Z. & Storm, D. R. The role of calmodulin as a signal integrator for synaptic plasticity. *Nat Rev Neurosci* **6,** 267–76 (2005).
38. Feng, B. & Stemmer, P. M. Interactions of calcineurin A, calcineurin B, and Ca2+. *Biochemistry (Mosc.)* **38,** 12481–9 (1999).
39. Shen, X. *et al.* The secondary structure of calcineurin regulatory region and conformational change induced by calcium/calmodulin binding. *J Biol Chem* **283,** 11407–13 (2008).
40. Gallagher, S. C. *et al.* There is communication between all four Ca(2+)-bindings sites of calcineurin B. *Biochemistry (Mosc.)* **40,** 12094–102 (2001).
41. O'Donnell, S. E., Yu, L., Fowler, C. A. & Shea, M. A. Recognition of β-calcineurin by the domains of calmodulin: thermodynamic and structural evidence for distinct roles. *Proteins* **79,** 765–86 (2011).

42. Quintana, A. R., Wang, D., Forbes, J. E. & Waxham, M. N. Kinetics of calmodulin binding to calcineurin. *Biochem Biophys Res Commun* **334,** 674–80 (2005).
43. Grabarek, Z. Structure of a trapped intermediate of calmodulin: calcium regulation of EF-hand proteins from a new perspective. *J Mol Biol* **346,** 1351–66 (2005).
44. Johnson, J. D., Snyder, C., Walsh, M. & Flynn, M. Effects of myosin light chain kinase and peptides on Ca2+ exchange with the N- and C-terminal Ca2+ binding sites of calmodulin. *J Biol Chem* **271,** 761–7 (1996).
45. Stratton, M. M., Chao, L. H., Schulman, H. & Kuriyan, J. Structural studies on the regulation of Ca2+/calmodulin dependent protein kinase II. *Curr Opin Struct Biol* **23,** 292–301 (2013).
46. Shifman, J. M., Choi, M. H., Mihalas, S., Mayo, S. L. & Kennedy, M. B. Ca2+/calmodulin-dependent protein kinase II (CaMKII) is activated by calmodulin with two bound calciums. *Proc Natl Acad Sci U A* **103,** 13968–73 (2006).
47. Evans, T. I. & Shea, M. A. Energetics of calmodulin domain interactions with the calmodulin binding domain of CaMKII. *Proteins* **76,** 47–61 (2009).
48. Coultrap, S. J., Buard, I., Kulbe, J. R., Dell'Acqua, M. L. & Bayer, K. U. CaMKII autonomy is substrate-dependent and further stimulated by Ca2+/calmodulin. *J Biol Chem* **285,** 17930–7 (2010).
49. Byrne, M. J., Putkey, J. A., Waxham, M. N. & Kubota, Y. Dissecting cooperative calmodulin binding to CaM kinase II: a detailed stochastic model. *J Comput Neurosci* **27,** 621–38 (2009).
50. Bhalla, U. S. & Iyengar, R. Emergent properties of networks of biological signaling pathways. *Science* **283,** 381–7 (1999).
51. Jama, A. M. *et al.* Lobe-specific functions of Ca2+·calmodulin in alphaCa2+·calmodulin-dependent protein kinase II activation. *J Biol Chem* **286,** 12308–16 (2011).
52. Singla, S. I., Hudmon, A., Goldberg, J. M., Smith, J. L. & Schulman, H. Molecular characterization of calmodulin trapping by calcium/calmodulin-dependent protein kinase II. *J Biol Chem* **276,** 29353–60 (2001).
53. Cheng, D. *et al.* Relative and absolute quantification of postsynaptic density proteome isolated from rat forebrain and cerebellum. *Mol Cell Proteomics* **5,** 1158–70 (2006).
54. Lee, S. J., Escobedo-Lozoya, Y., Szatmari, E. M. & Yasuda, R. Activation of CaMKII in single dendritic spines during long-term potentiation. *Nature* **458,** 299–304 (2009).

55. Strack, S., Barban, M. A., Wadzinski, B. E. & Colbran, R. J. Differential inactivation of postsynaptic density-associated and soluble Ca2+/calmodulin-dependent protein kinase II by protein phosphatases 1 and 2A. *J Neurochem* **68,** 2119–28 (1997).
56. Bradshaw, J. M., Kubota, Y., Meyer, T. & Schulman, H. An ultrasensitive Ca2+/calmodulin-dependent protein kinase II-protein phosphatase 1 switch facilitates specificity in postsynaptic calcium signaling. *Proc Natl Acad Sci U A* **100,** 10512–7 (2003).
57. Illario, M. *et al.* Calcium/calmodulin-dependent protein kinase II binds to Raf-1 and modulates integrin-stimulated ERK activation. *J Biol Chem* **278,** 45101–8 (2003).
58. Salzano, M. *et al.* Calcium/calmodulin-dependent protein kinase II (CaMKII) phosphorylates Raf-1 at serine 338 and mediates Ras-stimulated Raf-1 activation. *Cell Cycle* **11,** 2100–6 (2012).
59. Harris, K. M. & Stevens, J. K. Dendritic spines of rat cerebellar Purkinje cells: serial electron microscopy with reference to their biophysical characteristics. *J Neurosci* **8,** 4455–69 (1988).
60. Czöndör, K. *et al.* Unified quantitative model of AMPA receptor trafficking at synapses. *Proc Natl Acad Sci U A* **109,** 3522–7 (2012).
61. Liu, J. P., Sim, A. T. & Robinson, P. J. Calcineurin inhibition of dynamin I GTPase activity coupled to nerve terminal depolarization. *Science* **265,** 970–3 (1994).
62. Anggono, V. *et al.* Syndapin I is the phosphorylation-regulated dynamin I partner in synaptic vesicle endocytosis. *Nat Neurosci* **9,** 752–60 (2006).
63. Beattie, E. C. *et al.* Regulation of AMPA receptor endocytosis by a signaling mechanism shared with LTD. *Nat Neurosci* **3,** 1291–300 (2000).
64. Belmeguenai, A. & Hansel, C. A role for protein phosphatases 1, 2A, and 2B in cerebellar long-term potentiation. *J Neurosci* **25,** 10768–72 (2005).
65. Pearson, G. *et al.* Mitogen-activated protein (MAP) kinase pathways: regulation and physiological functions. *Endocr Rev* **22,** 153–83 (2001).
66. Kakiuchi, S. *et al.* Quantitative determinations of calmodulin in the supernatant and particulate fractions of mammalian tissues. *J Biochem* **92,** 1041–8 (1982).
67. Nalefski, E. A. & Newton, A. C. Membrane binding kinetics of protein kinase C betaII mediated by the C2 domain. *Biochemistry (Mosc.)* **40,** 13216–29 (2001).
68. Pérez-Lara, A., Egea-Jiménez, A. L., Ausili, A., Corbalán-García, S. & Gómez-Fernández, J. C. The membrane binding kinetics of full-length PKCα is determined by membrane lipid composition. *Biochim Biophys Acta* **1821,** 1434–42 (2012).

Supplementary Table S1: Parameters of the model

ID	Species/Reactions	Parameters	Notes	Ref.
	Volume of the compartment	0.06e-15 L	We subtracted the volume of the endoplasmic reticulum to calculate the volume of the compartment simulated	[59]
	Ca^{2+}	~ 50 $nmol.L^{-1}$	Basal concentration	[11]
	SERCA	60 molecules		[11]
	PMCA	10 molecules		[11]
	NCX	4 molecules		[11]
	PLA_2	0.4 $\mu mol.L^{-1}$		[11]
	PKC	1 $\mu mol.L^{-1}$		[11]

	PP2A	1.5 µmol.L^{-1}		[11]
	Raf	0.15 µmol.L^{-1}		[65]
	RKIP	1.5 µmol.L^{-1}		This paper
	Raf-act	0.5 µmol.L^{-1}		[11]
	MEK	1.5 µmol.L^{-1}		[11]
	ERK	1 µmol.L^{-1}		[11]
	PP5	1 µmol.L^{-1}		[11]
	MKP	0.26 µmol.L^{-1}		[11]
	CaM	19 µmol.L^{-1}		[66]
	CaN	1 µmol.L^{-1}		This paper
	αCaMKII	250 molecules		Estimated from [53]

	EP	1 $\mu mol.L^{-1}$		This paper
	Synaptic AMPAR	~97 molecules		
	Extra-synaptic AMPAR	30 molecules		This paper
	Cytosolic AMPAR	73 molecules		This paper
	GRIP	200 molecules	Number of molecules estimated to sustain the population of $AMPAR_{syn}$ around 100 receptors	This paper
1	$Ca^{2+} + PMCA \underset{k_b}{\overset{k_f}{\rightleftharpoons}} (Ca^{2+})PMCA$ $(Ca^{2+})PMCA \xrightarrow{k_{cat}} PMCA$	$k_f = 2500\ \mu mol^{-1}.L.s^{-1}$ $k_b = 2000\ s^{-1}$ $k_{cat} = 125\ s^{-1}$		[11]

2	$Ca^{2+} + NCX \underset{k_b}{\overset{k_f}{\rightleftharpoons}} (Ca^{2+})NCX$<br>$(Ca^{2+})NCX \xrightarrow{k_{cat}} NCX$	k_f = 800 µmol^{-1}.L.s^{-1}<br>k_b = 100 s^{-1}<br>k_{cat} = 2300 s^{-1}		[11]
3	$Ca^{2+} + SERCA \underset{k_{b1}}{\overset{k_{f1}}{\rightleftharpoons}} (Ca^{2+})SERCA$<br>$Ca^{2+} + (Ca^{2+})SERCA \underset{k_{b2}}{\overset{k_{f2}}{\rightleftharpoons}} (Ca^{2+})_2 SERCA$<br>$(Ca^{2+})_2 SERCA \xrightarrow{k_{cat}} SERCA$	k_{f1} = k_{f2} =<br>17147 µmol^{-1}.L.s^{-1}<br>k_{b1} = k_{b2} = 8426.3 s^{-1}<br>k_{cat} = 250 s^{-1}		[11]
4	$\xrightarrow{k_{leak}} Ca^{2+}$	k_{leak}= 100 µmol.L^{-1}.s^{-1}	Constant zero-order reaction of Ca^{2+} leak to the cytosol to sustain the basal [Ca^{2+}]	This paper
5	$Ca^{2+} + PLA_2 \underset{k_b}{\overset{k_f}{\rightleftharpoons}} \left(Ca^{2+}\right) PLA_2$	k_f = 11 µmol^{-1}.L.s^{-1}<br>k_b = 110 s^{-1}	The phosphorylation of PLA_2 did not alter its rate constants for interactions with Ca^{2+}	[1,23]

6	$Ca^{2+} + \left(Ca^{2+}\right)PLA_2 \underset{k_b}{\overset{k_f}{\rightleftharpoons}} \left(Ca^{2+}\right)_2 PLA_2$	$k_f = 1.83\ \mu mol^{-1}.L.s^{-1}$<br>$k_b = 110\ s^{-1}$		[1,23]
7	$Ca^{2+} + PLA_{2\,memb} \underset{k_b}{\overset{k_f}{\rightleftharpoons}} \left(Ca^{2+}\right)PLA_{2\,memb}$	$k_f = 11\ \mu mol^{-1}.L.s^{-1}$<br>$k_b = 2.5\ s^{-1}$		[1,23]
8	$Ca^{2+} + \left(Ca^{2+}\right)PLA_{2\,memb} \underset{k_b}{\overset{k_f}{\rightleftharpoons}} \left(Ca^{2+}\right)_2 PLA_{2\,memb}$	$k_f = 1.83\ \mu mol^{-1}.L.s^{-1}$<br>$k_b = 0.41\ s^{-1}$		[1,23]
9	$\left(Ca^{2+}\right)_2 PLA_2 \underset{k_b}{\overset{k_f}{\rightleftharpoons}} \left(Ca^{2+}\right)_2 PLA_{2\,memb}$	$k_f = 26\ s^{-1}$<br>$k_b = 0.05\ s^{-1}$		[24,25]
	$\left(Ca^{2+}\right)PLA_2 \underset{k_b}{\overset{k_f}{\rightleftharpoons}} \left(Ca^{2+}\right)PLA_{2\,memb}$	$k_f = 26\ s^{-1}$<br>$k_b = 2.5\ s^{-1}$		This paper
10	$PLA_2 \underset{k_b}{\overset{k_f}{\rightleftharpoons}} PLA_{2\,memb}$	$k_f = 26\ s^{-1}$<br>$k_b = 260\ s^{-1}$		This paper
11	$\left(Ca^{2+}\right)_2 PLA_{2\,memb} \underset{k_b}{\overset{k_f}{\rightleftharpoons}} \left(Ca^{2+}\right)_2 PLA^{*}_{2\,memb} \xrightarrow{k_{cat}} \left(Ca^{2+}\right)_2 PLA_{2\,memb} + AA$	$k_f = 150\ s^{-1}$<br>$k_b = 600\ s^{-1}$<br>$k_{cat} = 450\ s^{-1}$	The term * indicates the complex enzyme substrate	[11]
12	$PLA^{P}_{2\,memb} \underset{k_b}{\overset{k_f}{\rightleftharpoons}} PLA^{P^*}_{2\,memb} \xrightarrow{k_{cat}} PLA^{P}_{2\,memb} + AA$	$k_f = 150\ s^{-1}$<br>$k_b = 600\ s^{-1}$<br>$k_{cat} = 900\ s^{-1}$	The term P indicates PLA_2 in its	[2,11,25]

			phosphorylated state	
13	$\left(Ca^{2+}\right)PLA^{P}_{2\ memb} \underset{k_b}{\overset{k_f}{\rightleftharpoons}} \left(Ca^{2+}\right)PLA^{P^*}_{2\ memb} \xrightarrow{k_{cat}} \left(Ca^{2+}\right)PLA^{P}_{2\ memb} + AA$	k_f = 150 s^{-1}<br>k_b = 600 s^{-1}<br>k_{cat} = 900 s^{-1}		[2,11,25]
14	$\left(Ca^{2+}\right)_2 PLA^{P}_{2\ memb} \underset{k_b}{\overset{k_f}{\rightleftharpoons}} \left(Ca^{2+}\right)_2 PLA^{P^*}_{2\ memb} \xrightarrow{k_{cat}} \left(Ca^{2+}\right)_2 PLA^{P}_{2\ memb} + AA$	k_f = 150 s^{-1}<br>k_b = 600 s^{-1}<br>k_{cat} = 900 s^{-1}		[2,11,25]
15	$AA \xrightarrow{k_{\deg}}$	k_{deg} = 0.4 s^{-1}	Degradation of AA	[11]
16	$\left(Ca^{2+}\right)_2 PLA^{P}_{2} \underset{k_b}{\overset{k_f}{\rightleftharpoons}} \left(Ca^{2+}\right)_2 PLA^{P}_{2\ memb}$	k_f = 260 s^{-1}<br>k_b = 0.05 s^{-1}		[24,25]
	$PLA^{P}_{2} \underset{k_b}{\overset{k_f}{\rightleftharpoons}} PLA^{P}_{2\ memb}$	k_f = 260 s^{-1}<br>k_b = 260 s^{-1}		This paper
17	$\left(Ca^{2+}\right)PLA^{P}_{2} \underset{k_b}{\overset{k_f}{\rightleftharpoons}} \left(Ca^{2+}\right)PLA^{P}_{2\ memb}$	k_f = 260 s^{-1}<br>k_b = 2.5 s^{-1}		This paper
18	$PP + PLA^{P}_{2} \underset{k_b}{\overset{k_f}{\rightleftharpoons}} PP.PLA^{P}_{2} \xrightarrow{k_{cat}} PP + PLA_2$	k_f = 1.4 $\mu mol^{-1}.L.s^{-1}$<br>k_b = 1.5 s^{-1}<br>k_{cat} = 2.5 s^{-1}	PP states for PP2A or PP1	[11]

19	$PP + \left(Ca^{2+}\right)PLA_2^P \underset{k_b}{\overset{k_f}{\rightleftharpoons}} PP.\left(Ca^{2+}\right)PLA_2^P \xrightarrow{k_{cat}} PP + \left(Ca^{2+}\right)PLA_2$	k_f = 1.4 µmol^{-1}.L.s^{-1}<br>k_b = 1.5 s^{-1}<br>k_{cat} = 2.5 s^{-1}		[11]
20	$PP + \left(Ca^{2+}\right)_2 PLA_2^P \underset{k_b}{\overset{k_f}{\rightleftharpoons}} PP.\left(Ca^{2+}\right)_2 PLA_2^P \xrightarrow{k_{cat}} PP + \left(Ca^{2+}\right)_2 PLA_2$	k_f = 1.4 µmol^{-1}.L.s^{-1}<br>k_b = 1.5 s^{-1}<br>k_{cat} = 2.5 s^{-1}		[11]
21	$PP + \left(Ca^{2+}\right)PLA^P_{2\ memb} \underset{k_b}{\overset{k_f}{\rightleftharpoons}} PP.\left(Ca^{2+}\right)PLA_{2\ memb} \xrightarrow{k_{cat}} PP + \left(Ca^{2+}\right)PLA_{2\ memb}$	k_f = 1.4 µmol^{-1}.L.s^{-1}<br>k_b = 1.5 s^{-1}<br>k_{cat} = 2.5 s^{-1}		[11]
22	$PP + \left(Ca^{2+}\right)_2 PLA^P_{2\ memb} \underset{k_b}{\overset{k_f}{\rightleftharpoons}} PP.\left(Ca^{2+}\right)_2 PLA_{2\ memb} \xrightarrow{k_{cat}} PP + \left(Ca^{2+}\right)_2 PLA_{2\ memb}$	k_f = 1.4 µmol^{-1}.L.s^{-1}<br>k_b = 1.5 s^{-1}<br>k_{cat} = 2.5 s^{-1}		[11]
23	$PKC \underset{k_b}{\overset{k_f}{\rightleftharpoons}} PKC_{memb}$	k_f = 420 s^{-1}<br>k_b = 8400 s^{-1}		[28]
24	$Ca^{2+} + PKC \underset{k_b}{\overset{k_f}{\rightleftharpoons}} \left(Ca^{2+}\right)PKC$	k_f = 1111 µmol^{-1}.L.s^{-1}<br>k_b = 1000 s^{-1}	The same rate constants were used to simulate the interaction between Ca^{2+}	[11,29]

			and PKC bound to AA	
25	$Ca^{2+} + \left(Ca^{2+}\right) PKC \underset{k_b}{\overset{k_f}{\rightleftharpoons}} \left(Ca^{2+}\right)_2 PKC$	k_f = 45.45 µmol^{-1}.L.s^{-1}<br>k_b = 1000 s^{-1}		[11,29]
26	$Ca^{2+} + \left(Ca^{2+}\right)_2 PKC \underset{k_b}{\overset{k_f}{\rightleftharpoons}} \left(Ca^{2+}\right)_3 PKC$	k_f = 45.45 µmol^{-1}.L.s^{-1}<br>k_b = 1000 s^{-1}		[11,29]
27	$Ca^{2+} + PKC_{memb} \underset{k_b}{\overset{k_f}{\rightleftharpoons}} \left(Ca^{2+}\right) PKC_{memb}$	k_f = 1111 µmol^{-1}.L.s^{-1}<br>k_b = 12 s^{-1}		[11,28,29]
28	$Ca^{2+} + \left(Ca^{2+}\right) PKC_{memb} \underset{k_b}{\overset{k_f}{\rightleftharpoons}} \left(Ca^{2+}\right)_2 PKC_{memb}$	k_f = 45.45 µmol^{-1}.L.s^{-1}<br>k_b = 40 s^{-1}		[11,28,29]
29	$Ca^{2+} + \left(Ca^{2+}\right)_2 PKC_{memb} \underset{k_b}{\overset{k_f}{\rightleftharpoons}} \left(Ca^{2+}\right)_3 PKC_{memb}$	k_f = 45.45 µmol^{-1}.L.s^{-1}<br>k_b = 40 s^{-1}		[11,28,29]
30	$\left(Ca^{2+}\right) PKC \underset{k_b}{\overset{k_f}{\rightleftharpoons}} \left(Ca^{2+}\right) PKC_{memb}$	k_f = 420 s^{-1}<br>k_b = 4200 s^{-1}		[11,24]
31	$\left(Ca^{2+}\right)_2 PKC \underset{k_b}{\overset{k_f}{\rightleftharpoons}} \left(Ca^{2+}\right)_2 PKC_{memb}$	k_f = 420 s^{-1}<br>k_b = 0.017 s^{-1}		[11,24]
32	$\left(Ca^{2+}\right)_3 PKC \underset{k_b}{\overset{k_f}{\rightleftharpoons}} \left(Ca^{2+}\right)_3 PKC_{memb}$	k_f = 420 s^{-1}<br>k_b = 0.0017 s^{-1}		[11,24]
33	$AA + PKC \underset{k_b}{\overset{k_f}{\rightleftharpoons}} AA.PKC$	k_f = 1 µmol^{-1}.L.s^{-1}<br>k_b = 10 s^{-1}	These rates were kept unchanged	[11]

			for the interactions between AA and PKC bound to Ca^{2+} and/or membrane	
34	$AA.PKC \underset{k_b}{\overset{k_f}{\rightleftharpoons}} AA.PKC_{memb}$	$k_f = 420\ s^{-1}$<br>$k_b = 420\ s^{-1}$		This paper
35	$AA.\left(Ca^{2+}\right)PKC \underset{k_b}{\overset{k_f}{\rightleftharpoons}} AA.\left(Ca^{2+}\right)PKC_{memb}$	$k_f = 420\ s^{-1}$<br>$k_b = 42\ s^{-1}$		This paper
36	$AA.\left(Ca^{2+}\right)_2 PKC \underset{k_b}{\overset{k_f}{\rightleftharpoons}} AA.\left(Ca^{2+}\right)_2 PKC_{memb}$	$k_f = 420\ s^{-1}$<br>$k_b = 0.00017\ s^{-1}$		This paper
37	$AA.\left(Ca^{2+}\right)_3 PKC \underset{k_b}{\overset{k_f}{\rightleftharpoons}} AA.\left(Ca^{2+}\right)_3 PKC_{memb}$	$k_f = 420\ s^{-1}$<br>$k_b = 0.000017\ s^{-1}$		This paper
38	$RKIP + Raf \underset{k_b}{\overset{k_f}{\rightleftharpoons}} RKIP.Raf$<br>$RKIP + Raf^P \underset{k_b}{\overset{k_f}{\rightleftharpoons}} RKIP.Raf^P$	$k_f = 1\mu mol^{-1}.L.s^{-1}$<br>$k_b = 1\ s^{-1}$		[31]
39	$AA.\left(Ca^{2+}\right)_3 PKC_{memb} + RKIP \underset{k_b}{\overset{k_f}{\rightleftharpoons}} AA.\left(Ca^{2+}\right)_3 PKC_{memb}.RKIP$<br>$AA.\left(Ca^{2+}\right)_3 PKC_{memb}.RKIP \xrightarrow{k_{cat}} AA.\left(Ca^{2+}\right)_3 PKC_{memb} + RKIP^P$	$k_f = 2.632\ \mu mol^{-1}.L.s^{-1}$<br>$k_b = 30\ s^{-1}$<br>$k_{cat} = 120\ s^{-1}$		Estimated

				from [32]
40	$AA.\left(Ca^{2+}\right)_2 PKC_{memb} + RKIP \underset{k_b}{\overset{k_f}{\rightleftharpoons}} AA.\left(Ca^{2+}\right)_2 PKC_{memb}.RKIP$<br>$AA.\left(Ca^{2+}\right)_2 PKC_{memb}.RKIP \xrightarrow{k_{cat}} AA.\left(Ca^{2+}\right)_2 PKC_{memb} + RKIP^P$	k_f = 2.632 μmol^{-1}.L.s^{-1}<br>k_b = 30 s^{-1}<br>k_{cat} = 120 s^{-1}		Estimated from [32]
41	$AA.\left(Ca^{2+}\right) PKC_{memb} + RKIP \underset{k_b}{\overset{k_f}{\rightleftharpoons}} AA.\left(Ca^{2+}\right) PKC_{memb}.RKIP$<br>$AA.\left(Ca^{2+}\right) PKC_{memb}.RKIP \xrightarrow{k_{cat}} AA.\left(Ca^{2+}\right) PKC_{memb} + RKIP^P$	k_f = 2.632 μmol^{-1}.L.s^{-1}<br>k_b = 30 s^{-1}<br>k_{cat} = 120 s^{-1}		Estimated from [32]
42	$AA.PKC_{memb} + RKIP \underset{k_b}{\overset{k_f}{\rightleftharpoons}} AA.PKC_{memb}.RKIP$<br>$AA.PKC_{memb}.RKIP \xrightarrow{k_{cat}} AA.PKC_{memb} + RKIP^P$	k_f = 2.632 μmol^{-1}.L.s^{-1}<br>k_b = 30 s^{-1}<br>k_{cat} = 120 s^{-1}		Estimated from [32]
43	$\left(Ca^{2+}\right)_3 PKC_{memb} + RKIP \underset{k_b}{\overset{k_f}{\rightleftharpoons}} \left(Ca^{2+}\right)_3 PKC_{memb}.RKIP$<br>$\left(Ca^{2+}\right)_3 PKC_{memb}.RKIP \xrightarrow{k_{cat}} \left(Ca^{2+}\right)_3 PKC_{memb} + RKIP^P$	k_f = 2.632 μmol^{-1}.L.s^{-1}<br>k_b = 30 s^{-1}<br>k_{cat} = 120 s^{-1}		Estimated from [32]
44	$RKIP^P \xrightarrow{k_{dephos}} RKIP$	k_{dephos} = 30 s^{-1}		[33]

45	$RKIP^P + Raf \underset{k_b}{\overset{k_f}{\rightleftharpoons}} RKIP^P.Raf$<br>$RKIP^P + Raf^P \underset{k_b}{\overset{k_f}{\rightleftharpoons}} RKIP^P.Raf^P$	k_f = 1µmol^{-1}.L.s^{-1}<br>k_b = 100 s^{-1}		This paper, estimated from [31]
46	$AA.(Ca^{2+})_3 PKC_{memb} + Raf-act \underset{k_b}{\overset{k_f}{\rightleftharpoons}} AA.(Ca^{2+})_3 PKC_{memb}.Raf-act$<br>$AA.(Ca^{2+})_3 PKC_{memb}.Raf-act \xrightarrow{k_{cat}} AA.(Ca^{2+})_3 PKC_{memb} + Raf-act^P$	k_f =5.8 µmol^{-1}.L.s^{-1}<br>k_b = 0.56 s^{-1}<br>k_{cat} = 0.14 s^{-1}		[11]
47	$AA.(Ca^{2+})_2 PKC_{memb} + Raf-act \underset{k_b}{\overset{k_f}{\rightleftharpoons}} AA.(Ca^{2+})_2 PKC_{memb}.Raf-act$<br>$AA.(Ca^{2+})_2 PKC_{memb}.Raf-act \xrightarrow{k_{cat}} AA.(Ca^{2+})_2 PKC_{memb} + Raf-act^P$	k_f =5.8 µmol^{-1}.L.s^{-1}<br>k_b = 3.608 s^{-1}<br>k_{cat} = 4.7 s^{-1}		[11]
48	$AA.(Ca^{2+}) PKC_{memb} + Raf-act \underset{k_b}{\overset{k_f}{\rightleftharpoons}} AA.(Ca^{2+}) PKC_{memb}.Raf-act$<br>$AA.(Ca^{2+}) PKC_{memb}.Raf-act \xrightarrow{k_{cat}} AA.(Ca^{2+}) PKC_{memb} + Raf-act^P$	k_f =5.8 µmol^{-1}.L.s^{-1}<br>k_b = 3.608 s^{-1}<br>k_{cat} = 4.7 s^{-1}		[11]
49	$AA.PKC_{memb} + Raf-act \underset{k_b}{\overset{k_f}{\rightleftharpoons}} AA.PKC_{memb}.Raf-act$<br>$AA.PKC_{memb}.Raf-act \xrightarrow{k_{cat}} AA.PKC_{memb} + Raf-act^P$	k_f =5.8 µmol^{-1}.L.s^{-1}<br>k_b = 3.608 s^{-1}<br>k_{cat} = 4.7 s^{-1}		[11]

50	$\left(Ca^{2+}\right)_3 PKC_{memb} + Raf-act \underset{k_b}{\overset{k_f}{\rightleftharpoons}} \left(Ca^{2+}\right)_3 PKC_{memb}.Raf-act$<br>$\left(Ca^{2+}\right)_3 PKC_{memb}.Raf-act \xrightarrow{k_{cat}} \left(Ca^{2+}\right)_3 PKC_{memb} + Raf-act^P$	k_f =5.8 µmol^{-1}.L.s^{-1}<br>k_b = 3.608 s^{-1}<br>k_{cat} = 4.7 s^{-1}		11
51	$Raf-act^P \xrightarrow{k_{dephos}} Raf-act$	k_{dephos} = 1 s^{-1}		11
52	$Raf-act + Raf \underset{k_b}{\overset{k_f}{\rightleftharpoons}} Raf-act.Raf$<br>$Raf-act.Raf \xrightarrow{k_{cat}} Raf-act + Raf^P$	k_f = 1 µmol^{-1}.L.s^{-1}<br>k_b = 2 s^{-1}<br>k_{cat} = 1.5 s^{-1}		11
53	$PP5 + Raf^P \underset{k_b}{\overset{k_f}{\rightleftharpoons}} PP5.Raf^P$<br>$PP5.Raf^P \xrightarrow{k_{cat}} PP5 + Raf$	k_f = 0.55 µmol^{-1}.L.s^{-1}<br>k_b = 2 s^{-1}<br>k_{cat} = 0.5 s^{-1}		11
54	$Raf^P + MEK \underset{k_b}{\overset{k_f}{\rightleftharpoons}} Raf^P.MEK$<br>$Raf^P.MEK \xrightarrow{k_{cat}} Raf^P + MEK^P$<br>$Raf^P + MEK^P \underset{k_b}{\overset{k_f}{\rightleftharpoons}} Raf^P.MEK^P$<br>$Raf^P.MEK^P \xrightarrow{k_{cat}} Raf^P + MEK^{PP}$	k_f = 0.65 µmol^{-1}.L.s^{-1}<br>k_b = 0.065 s^{-1}<br>k_{cat} = 1 s^{-1}	The term ^{P} and ^{PP} indicate phosphorylated and double phosphorylated state, respectively	11

55	$PP2A + MEK^{PP} \underset{k_b}{\overset{k_f}{\rightleftharpoons}} PP2A.MEK^{PP}$<br>$PP2A.MEK^{PP} \xrightarrow{k_{cat}} PP2A + MEK^{P}$<br>$PP2A + MEK^{P} \underset{k_b}{\overset{k_f}{\rightleftharpoons}} PP2A.MEK^{P}$<br>$PP2A.MEK^{P} \xrightarrow{k_{cat}} PP2A + MEK$	k_f = 0.75 µmol^{-1}.L.s^{-1}<br>k_b = 2 s^{-1}<br>k_{cat} = 0.5 s^{-1}		[11]
56	$MEK^{PP} + ERK \underset{k_b}{\overset{k_f}{\rightleftharpoons}} MEK^{PP}.ERK$<br>$MEK^{PP}.ERK \xrightarrow{k_{cat}} MEK^{PP} + ERK^{P}$	k_f = 16.2 µmol^{-1}.L.s^{-1}<br>k_b = 0.6 s^{-1}<br>k_{cat} = 0.15 s^{-1}		[11]
57	$MEK^{PP} + ERK^{P} \underset{k_b}{\overset{k_f}{\rightleftharpoons}} MEK^{PP}.ERK^{P}$<br>$MEK^{PP}.ERK^{P} \xrightarrow{k_{cat}} MEK^{PP} + ERK^{PP}$	k_f = 16.2 µmol^{-1}.L.s^{-1}<br>k_b = 0.6 s^{-1}<br>k_{cat} = 0.3 s^{-1}		[11]
58	$MKP + ERK^{PP} \underset{k_b}{\overset{k_f}{\rightleftharpoons}} MKP.ERK^{PP}$<br>$MKP.ERK^{PP} \xrightarrow{k_{cat}} MKP + ERK^{P}$	k_f = 13 µmol^{-1}.L.s^{-1}<br>k_b = 0.396 s^{-1}<br>k_{cat} = 0.099 s^{-1}		[11]
59	$MKP + ERK^{P} \underset{k_b}{\overset{k_f}{\rightleftharpoons}} MKP.ERK^{P}$<br>$MKP.ERK^{P} \xrightarrow{k_{cat}} MKP + ERK$	k_f = 28 µmol^{-1}.L.s^{-1}<br>k_b = 0.56 s^{-1}<br>k_{cat} = 0.14 s^{-1}		[11]
60	$ERK^{PP} + PLA_2 \underset{k_b}{\overset{k_f}{\rightleftharpoons}} ERK^{PP}.PLA_2$<br>$ERK^{PP}.PLA_2 \xrightarrow{k_{cat}} ERK^{PP} + PLA_2^{P}$	k_f = 15.6 µmol^{-1}.L.s^{-1}<br>k_b = 56 s^{-1}<br>k_{cat} = 100 s^{-1}		Estim ated

				from [36]
61	$ERK^{PP} + \left(Ca^{2+}\right)PLA_2 \underset{k_b}{\overset{k_f}{\rightleftharpoons}} ERK^{PP}.\left(Ca^{2+}\right)PLA_2$<br>$ERK^{PP}.\left(Ca^{2+}\right)PLA_2 \xrightarrow{k_{cat}} ERK^{PP} + \left(Ca^{2+}\right)PLA_2^P$	k_f = 15.6 µmol^{-1}.L.s^{-1}<br>k_b = 56 s^{-1}<br>k_{cat} = 100 s^{-1}		Estimated from [36]
62	$ERK^{PP} + \left(Ca^{2+}\right)_2 PLA_2 \underset{k_b}{\overset{k_f}{\rightleftharpoons}} ERK^{PP}.\left(Ca^{2+}\right)_2 PLA_2$<br>$ERK^{PP}.\left(Ca^{2+}\right)_2 PLA_2 \xrightarrow{k_{cat}} ERK^{PP} + \left(Ca^{2+}\right)_2 PLA_2^P$	k_f = 15.6 µmol^{-1}.L.s^{-1}<br>k_b = 56 s^{-1}<br>k_{cat} = 100 s^{-1}		Estimated from [36]
63	$ERK^{PP} + \left(Ca^{2+}\right)PLA_{2_{memb}} \underset{k_b}{\overset{k_f}{\rightleftharpoons}} ERK^{PP}.\left(Ca^{2+}\right)PLA_{2_{memb}}$<br>$ERK^{PP}.\left(Ca^{2+}\right)PLA_{2_{memb}} \xrightarrow{k_{cat}} ERK^{PP} + \left(Ca^{2+}\right)PLA^P_{2\ memb}$	k_f = 15.6 µmol^{-1}.L.s^{-1}<br>k_b = 56 s^{-1}<br>k_{cat} = 100 s^{-1}		Estimated from [36]
64	$ERK^{PP} + \left(Ca^{2+}\right)_2 PLA_{2_{memb}} \underset{k_b}{\overset{k_f}{\rightleftharpoons}} ERK^{PP}.\left(Ca^{2+}\right)_2 PLA_{2_{memb}}$<br>$ERK^{PP}.\left(Ca^{2+}\right)_2 PLA_{2_{memb}} \xrightarrow{k_{cat}} ERK^{PP} + \left(Ca^{2+}\right)_2 PLA^P_{2\ memb}$	k_f = 15.6 µmol^{-1}.L.s^{-1}<br>k_b = 56 s^{-1}<br>k_{cat} = 100 s^{-1}		Estimated from [36]
65	$Ca^{2+} + CaM_{I,II} \underset{k_b}{\overset{k_f}{\rightleftharpoons}} (Ca^{2+})_I.CaM_{II}$	k_f = 750 µmol^{-1}.L.s^{-1}<br>k_b = 50000 s^{-1}	In the notation	[9]

			$(Ca^{2+})_xCaM_y$, the terms x and y indicate the Ca^{2+}-binding sites filled with Ca^{2+} and empty, respectively. Sites that were not declared explicitly did not interfere with the reactions described.	
66	$Ca^{2+} + CaM_{I,II} \underset{k_b}{\overset{k_f}{\rightleftharpoons}} (Ca^{2+})_{II}.CaM_I$	k_f = 750 µmol^{-1}.L.s^{-1}<br>k_b = 50000 s^{-1}		9
67	$Ca^{2+} + (Ca^{2+})_I.CaM_{II} \underset{k_b}{\overset{k_f}{\rightleftharpoons}} (Ca^{2+})_{I,II}.CaM$	k_f = 750 µmol^{-1}.L.s^{-1}<br>k_b = 625 s^{-1}		9
68	$Ca^{2+} + (Ca^{2+})_{II}.CaM_I \underset{k_b}{\overset{k_f}{\rightleftharpoons}} (Ca^{2+})_{I,II}.CaM$	k_f = 750 µmol^{-1}.L.s^{-1}<br>k_b = 625 s^{-1}		9

69	$Ca^{2+} + CaM_{III,IV} \underset{k_b}{\overset{k_f}{\rightleftharpoons}} (Ca^{2+})_{III}.CaM_{IV}$	k_f = 800 µmol^{-1}.L.s^{-1} k_b = 20000 s^{-1}		[9]
70	$Ca^{2+} + CaM_{III,IV} \underset{k_b}{\overset{k_f}{\rightleftharpoons}} (Ca^{2+})_{IV}.CaM_{III}$	k_f = 204 µmol^{-1}.L.s^{-1} k_b = 5115 s^{-1}		[9]
71	$Ca^{2+} + (Ca^{2+})_{III}.CaM_{IV} \underset{k_b}{\overset{k_f}{\rightleftharpoons}} (Ca^{2+})_{III,IV}.CaM$	k_f = 204 µmol^{-1}.L.s^{-1} k_b = 25.575 s^{-1}		[9]
72	$Ca^{2+} + (Ca^{2+})_{IV}.CaM_{III} \underset{k_b}{\overset{k_f}{\rightleftharpoons}} (Ca^{2+})_{III,IV}.CaM$	k_f = 800 µmol^{-1}.L.s^{-1} k_b = 100 s^{-1}		[9]
73	$Ca^{2+} + CNB_{I,II} \underset{k_b}{\overset{k_f}{\rightleftharpoons}} (Ca^{2+})_{I}.CNB_{II}$	k_f = 6.4 µmol^{-1}.L.s^{-1} k_b = 0.03 s^{-1}	The terms I, II, III and IV denote the Ca^{2+}-binding sites of CNB. In the notation $(Ca^{2+})_xCNB_y$, the terms x and y indicate the Ca^{2+}-binding sites filled with Ca^{2+} and empty, respectively.	[38,40]

			Sites that are not declared explicitly did not interfere with the reactions described.	
74	$Ca^{2+} + (Ca^{2+})_{I}.CNB_{II} \underset{k_b}{\overset{k_f}{\rightleftharpoons}} (Ca^{2+})_{I,II}.CNB$	k_f = 6.4 µmol^{-1}.L.s^{-1}<br>k_b = 0.0018 s^{-1}		38,40
75	$Ca^{2+} + CNB_{III,IV} \underset{k_b}{\overset{k_f}{\rightleftharpoons}} (Ca^{2+})_{III}.CNB_{IV}$	k_f = 0.09 µmol^{-1}.L.s^{-1}<br>k_b = 0.05 s^{-1}		38,40
76	$Ca^{2+} + (Ca^{2+})_{III}.CNB_{IV} \underset{k_b}{\overset{k_f}{\rightleftharpoons}} (Ca^{2+})_{III,IV}.CNB$	k_f = 0.09 µmol^{-1}.L.s^{-1}<br>k_b = 0.025 s^{-1}		38,40
77	$CNA + (Ca^{2+})_{N,C}.CaM \underset{k_b}{\overset{k_f}{\rightleftharpoons}} (Ca^{2+})_{N,C}.CaM.CNA$	k_f = 46 µmol^{-1}.L.s^{-1}<br>k_b = 0.0012 s^{-1}	The term $(Ca^{2+})_{N,C}$.CaM indicates that both the C and the N-terminal domains of CaM are associated with Ca^{2+}.	42

78	$CNA+(Ca^{2+})_C.CaM \underset{k_b}{\overset{k_f}{\rightleftharpoons}} (Ca^{2+})_C.CaM.CNA$	k_f = 46 µmol^{-1}.L.s^{-1} k_b = 46 s^{-1}	The term $(Ca^{2+})_C$.CaM indicates that only the C-terminal domain of CaM is associated with Ca^{2+}.	[41,42]
79	$CNA+(Ca^{2+})_N.CaM \underset{k_b}{\overset{k_f}{\rightleftharpoons}} (Ca^{2+})_N.CaM.CNA$	k_f = 46 µmol^{-1}.L.s^{-1} k_b = 322 s^{-1}	The term $(Ca^{2+})_N$.CaM indicates that only the N-terminal domain of CaM is associated with Ca^{2+}.	[41,42]
80	$Ca^{2+}+CaM_{I,II}.CNA \underset{k_b}{\overset{k_f}{\rightleftharpoons}} (Ca^{2+})_I.CaM_{II}.CNA$	k_f = 750 µmol^{-1}.L.s^{-1} k_b = 950 s^{-1}		[9,41]
81	$Ca^{2+}+CaM_{I,II}.CNA \underset{k_b}{\overset{k_f}{\rightleftharpoons}} (Ca^{2+})_{II}.CaM_I.CNA$	k_f = 750 µmol^{-1}.L.s^{-1} k_b = 950 s^{-1}		[9,41]

82	$Ca^{2+} + (Ca^{2+})_{I}.CaM_{II}.CNA \underset{k_b}{\overset{k_f}{\rightleftharpoons}} (Ca^{2+})_{I,II}.CaM.CNA$	k_f = 750 µmol^{-1}.L.s^{-1}<br>k_b = 11.9 s^{-1}		[9,41]
83	$Ca^{2+} + (Ca^{2+})_{II}.CaM_{I}.CNA \underset{k_b}{\overset{k_f}{\rightleftharpoons}} (Ca^{2+})_{I,II}.CaM.CNA$	k_f = 750 µmol^{-1}.L.s^{-1}<br>k_b = 11.9 s^{-1}		[9,41]
84	$Ca^{2+} + CaM_{III,IV}.CNA \underset{k_b}{\overset{k_f}{\rightleftharpoons}} (Ca^{2+})_{III}.CaM_{IV}.CNA$	k_f = 800 µmol^{-1}.L.s^{-1}<br>k_b = 160 s^{-1}		[9,41]
85	$Ca^{2+} + CaM_{III,IV}.CNA \underset{k_b}{\overset{k_f}{\rightleftharpoons}} (Ca^{2+})_{IV}.CaM_{III}.CNA$	k_f = 204 µmol^{-1}.L.s^{-1}<br>k_b = 48 s^{-1}		[9,41]
86	$Ca^{2+} + (Ca^{2+})_{III}.CaM_{IV}.CNA \underset{k_b}{\overset{k_f}{\rightleftharpoons}} (Ca^{2+})_{III,IV}.CaM.CNA$	k_f = 204 µmol^{-1}.L.s^{-1}<br>k_b = 0.24 s^{-1}		[9,41]
87	$Ca^{2+} + (Ca^{2+})_{IV}.CaM_{III}.CNA \underset{k_b}{\overset{k_f}{\rightleftharpoons}} (Ca^{2+})_{III,IV}.CaM.CNA$	k_f = 800 µmol^{-1}.L.s^{-1}<br>k_b = 0.8 s^{-1}		[9,41]
88	$\alpha CaMKII + (Ca^{2+})_{N,C}.CaM \underset{k_b}{\overset{k_f}{\rightleftharpoons}} (Ca^{2+})_{N,C}.CaM.\alpha CaMKII$	k_f = 20 µmol^{-1}.L.s^{-1}<br>k_b = 20 s^{-1}	The k_b was estimated in this paper. The term $Ca^{2+})_{N,C}$.CaM indicates that both the C and the N-terminal domains of CaM	[7,8]

			are bound to Ca^{2+}.	
89	$(Ca^{2+})_x.CaM.\alpha CaMKII + (Ca^{2+})_{N,C}.CaM \underset{k_b}{\overset{k_f}{\rightleftharpoons}}$ $(Ca^{2+})_{N,C}.CaM.(Ca^{2+})_x.CaM.\alpha CaMKII$	k_f = 20 µmol^{-1}.L.s^{-1} k_b = 18 s^{-1}	The k_b was estimated in this paper.	[7,8]
90	$\alpha CaMKII + (Ca^{2+})_C.CaM \underset{k_b}{\overset{k_f}{\rightleftharpoons}} (Ca^{2+})_C.CaM.\alpha CaMKII$	k_f = 20 µmol^{-1}.L.s^{-1} k_b = 5000 s^{-1}	The term $Ca^{2+})_C$.CaM indicates that only the C-terminal domain of CaM is associated to Ca^{2+}.	[7,8,46,47]
91	$(Ca^{2+})_x.CaM.\alpha CaMKII + (Ca^{2+})_C.CaM \underset{k_b}{\overset{k_f}{\rightleftharpoons}}$ $(Ca^{2+})_C.CaM.(Ca^{2+})_x.CaM.\alpha CaMKII$	k_f = 20 µmol^{-1}.L.s^{-1} k_b = 4500 s^{-1}	The k_b was estimated in this paper.	[7,8,46,47]
92	$Ca^{2+} + CaM_{I,II}.\alpha CaMKII \underset{k_b}{\overset{k_f}{\rightleftharpoons}} (Ca^{2+})_I.CaM_{II}.\alpha CaMKII$	k_f = 750 µmol^{-1}.L.s^{-1} k_b = 500 s^{-1}		[9,44,47]
93	$Ca^{2+} + CaM_{I,II}.\alpha CaMKII \underset{k_b}{\overset{k_f}{\rightleftharpoons}} (Ca^{2+})_{II}.CaM_I.\alpha CaMKII$	k_f = 750 µmol^{-1}.L.s^{-1} k_b = 500 s^{-1}		[9,44,47]

94	$Ca^{2+} + (Ca^{2+})_{I}.CaM_{II}.\alpha CaMKII \underset{k_b}{\overset{k_f}{\rightleftharpoons}} (Ca^{2+})_{I,II}.CaM.\alpha CaMKII$	k_f = 750 µmol^{-1}.L.s^{-1}<br>k_b = 6.25 s^{-1}		9,44,47
95	$Ca^{2+} + (Ca^{2+})_{II}.CaM_{I}.\alpha CaMKII \underset{k_b}{\overset{k_f}{\rightleftharpoons}} (Ca^{2+})_{I,II}.CaM.\alpha CaMKII$	k_f = 750 µmol^{-1}.L.s^{-1}<br>k_b = 6.25 s^{-1}		9,44,47
96	$Ca^{2+} + CaM_{III,IV}.\alpha CaMKII \underset{k_b}{\overset{k_f}{\rightleftharpoons}} (Ca^{2+})_{III}.CaM_{IV}.\alpha CaMKII$	k_f = 800 µmol^{-1}.L.s^{-1}<br>k_b = 1000 s^{-1}		9,44,47
97	$Ca^{2+} + CaM_{III,IV}.\alpha CaMKII \underset{k_b}{\overset{k_f}{\rightleftharpoons}} (Ca^{2+})_{IV}.CaM_{III}.\alpha CaMKII$	k_f = 204 µmol^{-1}.L.s^{-1}<br>k_b = 255.75 s^{-1}		9,44,47
98	$Ca^{2+} + (Ca^{2+})_{III}.CaM_{IV}.\alpha CaMKII \underset{k_b}{\overset{k_f}{\rightleftharpoons}} (Ca^{2+})_{III,IV}.CaM.\alpha CaMKII$	k_f = 204 µmol^{-1}.L.s^{-1}<br>k_b = 1.2788 s^{-1}		9,44,47
99	$Ca^{2+} + (Ca^{2+})_{IV}.CaM_{III}.\alpha CaMKII \underset{k_b}{\overset{k_f}{\rightleftharpoons}} (Ca^{2+})_{III,IV}.CaM.\alpha CaMKII$	k_f = 800 µmol^{-1}.L.s^{-1}<br>k_b = 5 s^{-1}		9,44,47
100	$\left[(Ca^{2+})_{I,II,III,IV}.CaM\right]_2.\alpha CaMKII^{*} \xrightarrow{k_{cat}} \left[(Ca^{2+})_{I,II,III,IV}.CaM\right]_2.\alpha CaMKII^{P}$	k_{cat} = 5 s^{-1}	Autophoshorylation of CaMKII. ^{P} indicates phosphorylated state.	51
101	$\alpha CaMKII^{P} + (Ca^{2+})_{N,C}.CaM \underset{k_b}{\overset{k_f}{\rightleftharpoons}} (Ca^{2+})_{N,C}.CaM.\alpha CaMKII^{P}$	k_f = 20 µmol^{-1}.L.s^{-1}<br>k_b = 0.02 s^{-1}		7,8,52

102	$(Ca^{2+})_x.CaM.\alpha CaMKII^P + (Ca^{2+})_{N,C}.CaM \underset{k_b}{\overset{k_f}{\rightleftharpoons}}$ $(Ca^{2+})_{N,C}.CaM.(Ca^{2+})_x.CaM.\alpha CaMKII^P$	$k_f = 20\ \mu mol^{-1}.L.s^{-1}$ $k_b = 0.018\ s^{-1}$		[7,8,52]
103	$\alpha CaMKII^P + (Ca^{2+})_C.CaM \underset{k_b}{\overset{k_f}{\rightleftharpoons}} (Ca^{2+})_C.CaM.\alpha CaMKII^P$	$k_f = 20\ \mu mol^{-1}.L.s^{-1}$ $k_b = 5\ s^{-1}$		[7,8,46,47,52]
104	$(Ca^{2+})_x.CaM.\alpha CaMKII^P + (Ca^{2+})_C.CaM \underset{k_b}{\overset{k_f}{\rightleftharpoons}}$ $(Ca^{2+})_C.CaM.(Ca^{2+})_x.CaM.\alpha CaMKII^P$	$k_f = 20\ \mu mol^{-1}.L.s^{-1}$ $k_b = 4.5\ s^{-1}$		[7,8,46,47,52]
105	$PP + \alpha CaMKII^P \underset{k_b}{\overset{[k_f}{\rightleftharpoons}} PP.\alpha CaMKII^P \xrightarrow{k_{cat}} PP + \alpha CaMKII$	$k_f = 6.4\ s^{-1}$ $k_b = 56\ s^{-1}$ $k_{cat} = 14\ s^{-1}$	PP states for PP1 or PP2A	[55,56]
106	$\left[(Ca^{2+})_{I,II,III,IV}.CaM)\right]_2.\alpha CaMKII^P + Raf \underset{k_b}{\overset{k_f}{\rightleftharpoons}}$ $\left[(Ca^{2+})_{I,II,III,IV}.CaM)\right]_2.\alpha CaMKII^P.Raf \xrightarrow{k_{cat}}$ $\left[(Ca^{2+})_{I,II,III,IV}.CaM)\right]_2.\alpha CaMKII^P + Raf^P$	$k_f = 20\ s^{-1}$ $k_b = 50\ s^{-1}$ $k_{cat} = 150\ s^{-1}$		This paper
107	$\left[(Ca^{2+})_x.CaM)\right]_y.\alpha CaMKII^P + Raf \underset{k_b}{\overset{k_f}{\rightleftharpoons}}$ $\left[(Ca^{2+})_x.CaM)\right]_y.\alpha CaMKII^P.Raf \xrightarrow{k_{cat}}$ $\left[(Ca^{2+})_x.CaM)\right]_y.\alpha CaMKII^P + Raf^P$	$k_f = 20\ s^{-1}$ $k_b = 50\ s^{-1}$ $k_{cat} = 30\ s^{-1}$		This paper
108	$\alpha CaMKII^P + Raf \underset{k_b}{\overset{k_f}{\rightleftharpoons}} \alpha CaMKII^P.Raf \xrightarrow{k_{cat}} \alpha CaMKII^P + Raf^P$	$k_f = 20\ s^{-1}$ $k_b = 50\ s^{-1}$		This paper

		k_{cat} = 30 s^{-1}		
109	$AMPAR_{syn} \underset{k_b}{\overset{k_f}{\rightleftharpoons}} AMPAR_{extra}$	k_f = 0.05 s^{-1}<br>k_b = 0.065 s^{-1}		[11,59,60]
110	$AMPAR_{syn}^{P} \underset{k_b}{\overset{k_f}{\rightleftharpoons}} AMPAR_{extra}^{P}$	k_f = 0.05 s^{-1}<br>k_b = 0.065 s^{-1}		[11,59,60]
111	$EP^P + AMPAR_{extra} \underset{k_b}{\overset{k_f}{\rightleftharpoons}} EP^P.AMPAR_{extra} \xrightarrow{k_{cat}} EP^P + AMPAR_{endo}$	k_f = 0.03 s^{-1}<br>k_b = 0.02 s^{-1}<br>k_{cat} = 1 s^{-1}		This paper
112	$EP^P + AMPAR_{extra}^{P} \underset{k_b}{\overset{k_f}{\rightleftharpoons}} EP^P.AMPAR_{extra}^{P} \xrightarrow{k_{cat}} EP^P + AMPAR_{endo}^{P}$	k_f = 0.12 s^{-1}<br>k_b = 0.02 s^{-1}<br>k_{cat} = 1 s^{-1}		This paper
113	$AMPAR_{endo} \xrightarrow{k_{exo}} AMPAR_{extra}$	k_{exo} = 0.005 s^{-1}		This paper
114	$GRIP + AMPAR_{syn} \underset{k_b}{\overset{k_f}{\rightleftharpoons}} GRIP.AMPAR_{syn}$	k_f = 0.5 s^{-1}<br>k_b = 0.5 s^{-1}		[11,60]
115	$GRIP + AMPAR_{syn}^{P} \underset{k_b}{\overset{k_f}{\rightleftharpoons}} GRIP.AMPAR_{syn}^{P}$	k_f = 0.5 s^{-1}<br>k_b = 3.5 s^{-1}		[11,60]
116	$AA.\left(Ca^{2+}\right)_3 PKC_{memb} + AMPAR_{syn} \underset{k_b}{\overset{k_f}{\rightleftharpoons}} AA.\left(Ca^{2+}\right)_3 PKC_{memb}.AMPAR_{syn}$<br>$AA.\left(Ca^{2+}\right)_3 PKC_{memb}.AMPAR_{syn} \xrightarrow{k_{cat}} AA.\left(Ca^{2+}\right)_3 PKC_{memb} + AMPAR_{syn}^{P}$	k_f = 0.8 $\mu mol^{-1}.L.s^{-1}$<br>k_b = 0.8 s^{-1}<br>k_{cat} = 5 s^{-1}		This paper

117	$AA.\left(Ca^{2+}\right)_2 PKC_{memb} + AMPAR_{syn} \xrightleftharpoons[k_b]{k_f} AA.\left(Ca^{2+}\right)_2 PKC_{memb}.AMPAR_{syn}$<br>$AA.\left(Ca^{2+}\right)_2 PKC_{memb}.AMPAR_{syn} \xrightarrow{k_{cat}} AA.\left(Ca^{2+}\right)_2 PKC_{memb} + AMPAR_{syn}^{P}$	k_f = 0.8 µmol^{-1}.L.s^{-1}<br>k_b = 0.8 s^{-1}<br>k_{cat} = 5 s^{-1}		This paper
118	$AA.\left(Ca^{2+}\right) PKC_{memb} + AMPAR_{syn} \xrightleftharpoons[k_b]{k_f} AA.\left(Ca^{2+}\right) PKC_{memb}.AMPAR_{syn}$<br>$AA.\left(Ca^{2+}\right) PKC_{memb}.AMPAR_{syn} \xrightarrow{k_{cat}} AA.\left(Ca^{2+}\right) PKC_{memb} + AMPAR_{syn}^{P}$	k_f = 0.8 µmol^{-1}.L.s^{-1}<br>k_b = 0.8 s^{-1}<br>k_{cat} = 5 s^{-1}		This paper
119	$AA.PKC_{memb} + AMPAR_{syn} \xrightleftharpoons[k_b]{k_f} AA.PKC_{memb}.AMPAR_{syn}$<br>$AA.PKC_{memb}.AMPAR_{syn} \xrightarrow{k_{cat}} AA.PKC_{memb} + AMPAR_{syn}^{P}$	k_f = 0.8 µmol^{-1}.L.s^{-1}<br>k_b = 0.8 s^{-1}<br>k_{cat} = 5 s^{-1}		This paper
120	$\left(Ca^{2+}\right)_3 PKC_{memb} + AMPAR_{syn} \xrightleftharpoons[k_b]{k_f} \left(Ca^{2+}\right)_3 PKC_{memb} AMPAR_{syn}$<br>$\left(Ca^{2+}\right)_3 PKC_{memb}.AMPAR_{syn} \xrightarrow{k_{cat}} \left(Ca^{2+}\right)_3 PKC_{memb} + AMPAR_{syn}^{P}$	k_f = 0.8 µmol^{-1}.L.s^{-1}<br>k_b = 0.8 s^{-1}<br>k_{cat} = 5 s^{-1}		This paper
121	$PP2A + AMPAR_{syn}^{P} \xrightleftharpoons[k_b]{k_f} PP2A.AMPAR_{syn}^{P} \xrightarrow{k_{cat}} PP2A + AMPAR_{syn}$	k_f = 0.08 µmol^{-1}.L.s^{-1}<br>k_b = 0.8 s^{-1}<br>k_{cat} = 2 s^{-1}		This paper
122	$PP2A + AMPAR_{extra}^{P} \xrightleftharpoons[k_b]{k_f} PP2A.AMPAR_{extra}^{P} \xrightarrow{k_{cat}} PP2A + AMPAR_{extra}$	k_f = 0.08 µmol^{-1}.L.s^{-1}<br>k_b = 0.8 s^{-1}<br>k_{cat} = 2 s^{-1}		This paper
123	$PP2A + AMPAR_{endo}^{P} \xrightleftharpoons[k_b]{k_f} PP2A.AMPAR_{endo}^{P} \xrightarrow{k_{cat}} PP2A + AMPAR_{endo}$	k_f = 0.08 µmol^{-1}.L.s^{-1}<br>k_b = 0.8 s^{-1}		This paper

		k_{cat} = 2 s^{-1}		
124	$AA.\left(Ca^{2+}\right)_3 PKC_{memb} + EP \underset{k_b}{\overset{k_f}{\rightleftharpoons}} AA.\left(Ca^{2+}\right)_3 PKC_{memb}.EP$<br>$AA.\left(Ca^{2+}\right)_3 PKC_{memb}.EP \xrightarrow{k_{cat}} AA.\left(Ca^{2+}\right)_3 PKC_{memb} + EP^P$	k_f = 0.2 µmol^{-1}.L.s^{-1}<br>k_b = 0.8 s^{-1}<br>k_{cat} = 0.2 s^{-1}	The term ^{P} indicates phosphorylated state.	This paper
125	$AA.\left(Ca^{2+}\right)_2 PKC_{memb} + EP \underset{k_b}{\overset{k_f}{\rightleftharpoons}} AA.\left(Ca^{2+}\right)_2 PKC_{memb}.EP$<br>$AA.\left(Ca^{2+}\right)_2 PKC_{memb}.EP \xrightarrow{k_{cat}} AA.\left(Ca^{2+}\right)_2 PKC_{memb} + EP^P$	k_f = 0.2 µmol^{-1}.L.s^{-1}<br>k_b = 0.8 s^{-1}<br>k_{cat} = 0.2 s^{-1}		This paper
126	$AA.\left(Ca^{2+}\right) PKC_{memb} + EP \underset{k_b}{\overset{k_f}{\rightleftharpoons}} AA.\left(Ca^{2+}\right) PKC_{memb}.EP$<br>$AA.\left(Ca^{2+}\right) PKC_{memb}.EP \xrightarrow{k_{cat}} AA.\left(Ca^{2+}\right) PKC_{memb} + EP^P$	k_f = 0.2 µmol^{-1}.L.s^{-1}<br>k_b = 0.8 s^{-1}<br>k_{cat} = 0.2 s^{-1}		This paper
127	$AA.PKC_{memb} + EP \underset{k_b}{\overset{k_f}{\rightleftharpoons}} AA.PKC_{memb}.EP$<br>$AA.PKC_{memb}.EP \xrightarrow{k_{cat}} AA.PKC_{memb} + EP^P$	k_f = 0.2 µmol^{-1}.L.s^{-1}<br>k_b = 0.8 s^{-1}<br>k_{cat} = 0.2 s^{-1}		This paper
128	$\left(Ca^{2+}\right)_3 PKC_{memb} + EP \underset{k_b}{\overset{k_f}{\rightleftharpoons}} \left(Ca^{2+}\right)_3 PKC_{memb}EP$<br>$\left(Ca^{2+}\right)_3 PKC_{memb}.EP \xrightarrow{k_{cat}} \left(Ca^{2+}\right)_3 PKC_{memb} + EP^P$	k_f = 0.2 µmol^{-1}.L.s^{-1}<br>k_b = 0.8 s^{-1}<br>k_{cat} = 0.2 s^{-1}		This paper
129	$(Ca^{2+})_{N,C}.CaM.CNA + EP^P \underset{k_b}{\overset{k_f}{\rightleftharpoons}} (Ca^{2+})_{N,C}.CaM.CNA.EP^P$<br>$(Ca^{2+})_{N,C}.CaM.CNA.EP^P \xrightarrow{k_{cat}} (Ca^{2+})_{N,C}.CaM.CNA + EP$	k_f = 20 µmol^{-1}.L.s^{-1}<br>k_b = 50 s^{-1}<br>k_{cat} = 170 s^{-1}	Dephosphorylation of EP catalyzed by	This paper

			CaN bound to four Ca^{2+} and CaM fully saturated	
