# Supplementary material for: Stochastic Induction of Long-Term Potentiation and Long-Term Depression
Source: Sci Rep. 2016 Aug 3;6:30899. doi: 10.1038/srep30899 (PMC4971485; doi:10.1038/srep30899)
Supplement: Supplementary Dataset 1 [file srep30899-s2.zip › DataSet/README.pdf]

This is a MATLAB file (.mat) containing a structured array with the Dataset.

Please, type ‘load DataFiles.mat’ in the MATLAB command window to open the file. The fieldnames are organized with the name of the Figures (Fig1, Fig2, Fig3 and Fig5). Inside of each field there are other fieldnames for each figure panel (A, B, C, D, etc).

Then, there are fields Xdata and Ydata for the X and Y axis, respectively. There are Xdata and Ydata fields that contain a matrix (lines x columns) with all the conditions plotted in the same panel.

The DataSet.Fig5 contains the time course of AMPARs for each condition, and the respective calcium stimulus used to produce each point in Fig. 5. This data is the same as shown in the Supplementary Fig. S7.
